# *cis*-Golgi phosphate transporters harboring an EXS domain are essential for plant growth and development

**DOI:** 10.1093/plphys/kiad123

**Published:** 2023-02-28

**Authors:** Yi-Fang Hsieh, Dmitry Suslov, Luca Espen, Marion Schiavone, Carsten Rautengarten, Annika Griess-Osowski, Catalin Voiniciuc, Yves Poirier

**Affiliations:** Department of Plant Molecular Biology, University of Lausanne, Lausanne 1015, Switzerland; Department of Plant Physiology and Biochemistry, Faculty of Biology, Saint Petersburg State University, Saint Petersburg 199034, Russia; Department of Agricultural and Environmental Sciences—Production, Landscape, Agroenergy, Università degli Studi di Milano, Milan 20133, Italy; CNRS, Toulouse Biotechnology Institute, UMR 5504, Toulouse 31077, France; School of BioSciences, University of Melbourne, Parkville 3010, Victoria, Australia; Designer Glycans Group, Leibniz Institute of Plant Biochemistry, Halle 06120, Germany; Designer Glycans Group, Leibniz Institute of Plant Biochemistry, Halle 06120, Germany; Department of Plant Molecular Biology, University of Lausanne, Lausanne 1015, Switzerland

## Abstract

Cell wall synthesis and protein glycosylation require the import of nucleotide diphosphate–sugar conjugates into the Golgi that must be counterbalanced by phosphate (Pi) export. Numerous Golgi nucleotide-sugar transporters have been characterized, but transporters mediating Golgi Pi export remain poorly understood. We used plant and yeast genetics to characterize the role of 2 Arabidopsis (*Arabidopsis thaliana*) proteins possessing an EXS domain, namely ERD1A and ERD1B, in Golgi Pi homeostasis. ERD1A and ERD1B localized in *cis*-Golgi and were broadly expressed in vegetative and reproductive tissues. We identified ERD1 putative orthologs in algae, bryophytes, and vascular plants. Expressing *ERD1A* and *ERD1B* in yeast complemented the *erd1* mutant phenotype of cellular Pi loss via exocytosis associated with reduced Golgi Pi export. The Arabidopsis *erd1a* mutant had a similar phenotype of apoplastic Pi loss dependent on exocytosis. *ERD1A* overexpression in *Nicotiana benthamiana* and Arabidopsis led to partial mislocalization of ERD1A to the plasma membrane and specific Pi export to the apoplastic space. Arabidopsis *erd1a* had defects in cell wall biosynthesis, which were associated with reduced shoot development, hypocotyl growth, cell wall extensibility, root elongation, pollen germination, pollen tube elongation, and fertility. We identified ERD1 proteins as Golgi Pi exporters that are essential for optimal plant growth and fertility.

## Teaser

Import of nucleotide diphosphate–sugars into Golgi is balanced by phosphate export mediated by ERD1, a protein broadly conserved in plants and essential for optimal growth and development.

## Introduction

Phosphorus is one of the most limiting plant nutrients in natural and agricultural ecosystems. Plants acquire phosphorus from the environment as free inorganic phosphate (Pi), which is present at low concentrations in most soils (Poirier et al. 2022). Pi import into plants is primarily mediated by plasma membrane-localized H^+^-Pi co-transporters belonging to the phosphate transporter 1 (PHT1) family expressed in roots (Nussaume et al. 2011). Beyond the plasma membrane, transporter-mediated Pi flux across various membranes of organelles, such as plastids, mitochondria, and vacuoles, plays an important role in cellular Pi homeostasis (Liu et al. 2015, 2016; Poirier and Jung 2015; Versaw and Garcia 2017; Xu et al. 2019).

The Golgi system produces carbohydrate precursors for cell wall hemicellulose and pectin biosynthesis and for protein and lipid glycosylation (Verbancic et al. 2018; Zhang et al. 2021). Monosaccharides are imported into the Golgi as nucleotide diphosphate (NDP)–sugar conjugates. Once in the Golgi, the sugar moiety is released and the free NDP is de-phosphorylated by apyrases to give nucleotide monophosphate (NMP) and Pi (Chiu et al. 2015). The Golgi membrane contains numerous nucleotide sugar transporters (NSTs) that exchange cytosolic NDP-sugars for NMP from the Golgi lumen (Orellana et al. 2016). This exchange results in a net accumulation of sugars and Pi into the Golgi. Considering the large flux of NDP-sugars required for glycosylation and cell wall biosynthesis, a mechanism to export Pi out of the Golgi is needed to maintain cellular Pi homeostasis. Previous studies have identified Phosphate Transporter 4; 6 (PHT4;6) as a potential Pi exporter. PHT4;6 is distinct from other members of the PHT4 family in localizing to the *trans*-Golgi (Guo et al. 2008; Hassler et al. 2012). Heterologous expression of *PHT4;6* in yeast vacuoles led to Pi export, indicating that PHT4;6 transports Pi out of the Golgi in plants (Cubero et al. 2009). The Arabidopsis (*Arabidopsis thaliana*) *pht4;6* mutant has a strong rosette growth defect, altered senescence, changes in cell wall monosaccharide composition, and increased sensitivity to salt stress (Cubero et al. 2009; Hassler et al. 2012, 2016).

Phosphate 1 (PHO1) is a Pi exporter involved in Pi loading into the root xylem for Pi transport to the shoot (Poirier et al. 1991; Hamburger et al. 2002). PHO1 contains an N-terminal domain named SPX (from Syg1, Suppressor of Yeast G Protein 1/Pho81, Phosphate 81/Xpr1, Xenotropic Polytropic Retrovirus Receptor 1) involved in inositol pyrophosphate binding and a C-terminal domain named EXS (from ERD1, Endoplasmic Reticulum Defective 1/Xpr1/Syg1) shown to be essential for its Pi export activity (Wege et al. 2016; Wild et al. 2016; Jung et al. 2018). In addition to members of the *PHO1* family, the Arabidopsis genome contains 2 other genes, namely *At5g35730* and *At2g32295*, encoding EXS domain-containing proteins (Wang et al. 2004). The yeast *Saccharomyces cerevisiae* has 2 EXS domain-containing proteins: SYG1, which also harbors an SPX domain, and ERD1 (Spain et al. 1995; Wang et al. 2004). The yeast *erd1* mutant was initially characterized as deficient in luminal endoplasmic reticulum (ER) protein retention (Hardwick et al. 1990). An independent screen later reported that *erd1* has cell wall composition and/or structural defects, which affect its ability to bind Alcian blue dye; this phenotype may be associated with the mis-localization of Golgi glycosyltransferases (Corbacho et al. 2004; Okamoto et al. 2008; Sardana et al. 2021). Subsequently, yeast ERD1 was defined as a Golgi Pi exporter required to balance Pi import associated with NDP-sugar transport into the Golgi (Snyder et al. 2017). In the yeast *erd1* mutant, failure to export Golgi Pi to the cytosol resulted in increased apoplastic Pi export, which was dependent on the activity of the Golgi NDP-sugar transporter Vanadate-Resistant Glycosylation 4 (VRG4), as well as on exocytosis (Snyder et al. 2017).

In this work, we characterized the functions of the *A. thaliana* genes *ERD1A* (*At5g35730*) and *ERD1B* (*At2g32295*) in Pi homeostasis. We show that ERD1A and ERD1B are *cis*-Golgi proteins with overlapping functions in Pi transport that are distinct from the *trans*-Golgi PHT4;6, and that play important roles in plant growth and fertility.

## Results

### Arabidopsis ERD1A and ERD1B are broadly expressed in the *cis*-Golgi and have similar functions

Alignment of the *S. cerevisiae* ERD1 sequence with those of the Arabidopsis proteins encoded by *At5G35730* and *At2g32295* revealed 20.5% identity and 45.2% similarity between At5g35730 and ScERD1, and 18.1% identity and 40% similarity between At2g32295 and ScERD1 (Supplemental Fig. S1). In contrast, At5g35730 and At2g32295 showed 59.8% identity and 81.9% similarity with each other. The sequence similarity between ScERD1 and its Arabidopsis orthologs was higher in the carboxy half of the protein harboring the EXS domain. Therefore, we named *At5g35730* and *At2g32295* as *ERD1A* and *ERD1B*, respectively.

Phylogenic analysis revealed ERD1 putative orthologs in a broad spectrum of evolutionarily distant plants, including monocotyledons (*Zea mays*, *Triticum aestivum*, and *Oryza sativa*) and dicotyledons (*Medicago truncatula* and *Populus trichocarpa*), fern (*Ceratopteris richardii*), lycophyte (*Selaginella moellendorffii*), moss (*Physcomitrium patens*), liverwort (*Marchantia polymorpha*), and unicellular algae (*Ostreococcus tauri* and *Chlamydomonas reinhardtii*) (Supplemental Figs. S2 and S3, Supplemental Data Set S1).

**Figure 1.**
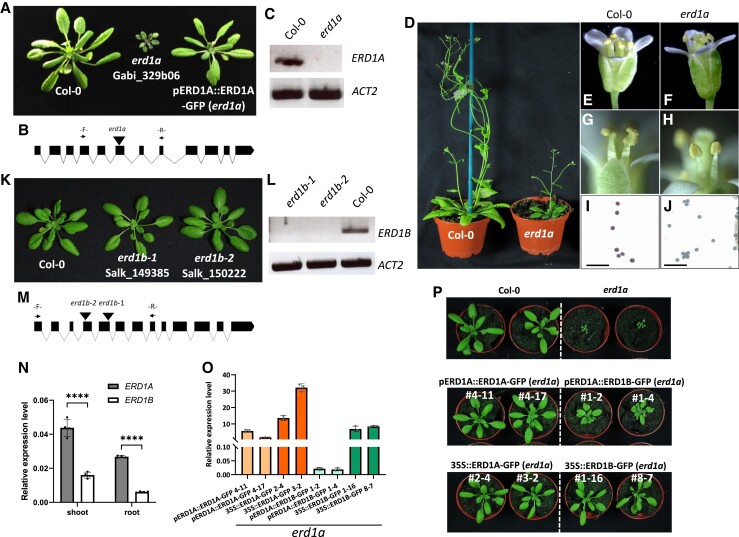
Phenotypes of *erd1a* and *erd1b* T-DNA mutants. Phenotypes of T-DNA insertion mutants in the *ERD1A***A)**, **D)** and *ERD1B***K)** genes are shown along with corresponding diagrams of the gene exon-intron structures with the location of the T-DNA insertion sites **B)**, **M)** and the RT-PCR analysis using oligos that span the insertion sites, showing disruption in gene expression **C)**, **L)**. The *erd1a* mutant phenotype was complemented by the *pERD1A::ERD1A-GFP* construct (**A**, right). In contrast to Col-0 **E)**, **G)**, **I)**, stamens of *erd1a* flowers failed to release mature pollen **F)**, **H)**, and Alexander's staining showed that *erd1a* pollen was not viable **J)**. For **I)** and **J)**, scale bars represent 100 *µ*m. **N)***ERD1A* and *ERD1B* expression level in roots and shoots of 10-d-old plants was measured by RT-qPCR. Student's *t*-test, error bars, ± Sd, *n* = 4, ****P < 0.0001. **O)** Expression of the *ERD1-GFP* and *ERD1B-GFP* transgenes was analyzed by RT-qPCR with primers targeting *GFP*. Expression is shown relative to *ACTIN2* (*ACT2*). Error bars, ± Sd, *n* ≥ 3. **P)** The *erd1a* mutant was complemented by expressing *ERD1A* via its native promoter (*pERD1A::ERD1A-GFP*) or the CaMV35S promoter (*35S::ERD1A-GFP*), or by expressing *ERD1B-GFP* with the CaMV35S promoter (*35S::ERD1B-GFP*). *ERD1B-GFP* expression via the *ERD1A* promoter (*pERD1A::ERD1B-GFP*) partially complemented the *erd1a* mutant (middle right). Four-week-old plants of 2 independent lines for each complementation are shown.

A homozygous mutant with a T-DNA insertion in the sixth exon of *ERD1A* (the *erd1a* mutant) that abolished its expression had stunted growth and was sterile due to the absence of viable pollen, as observed by Alexander's staining (Fig. 1, A–J). The *erd1a* rosette growth and male sterility phenotypes were complemented by expressing an *ERD1A-GFP* fusion construct under the control of the *ERD1A* promoter (Fig. 1A, right). In contrast, 2 independent T-DNA insertions in the fourth and fifth exons of *ERD1B* that abolished its expression did not lead to visible growth phenotypes (Fig. 1, K–M).

**Figure 2.**
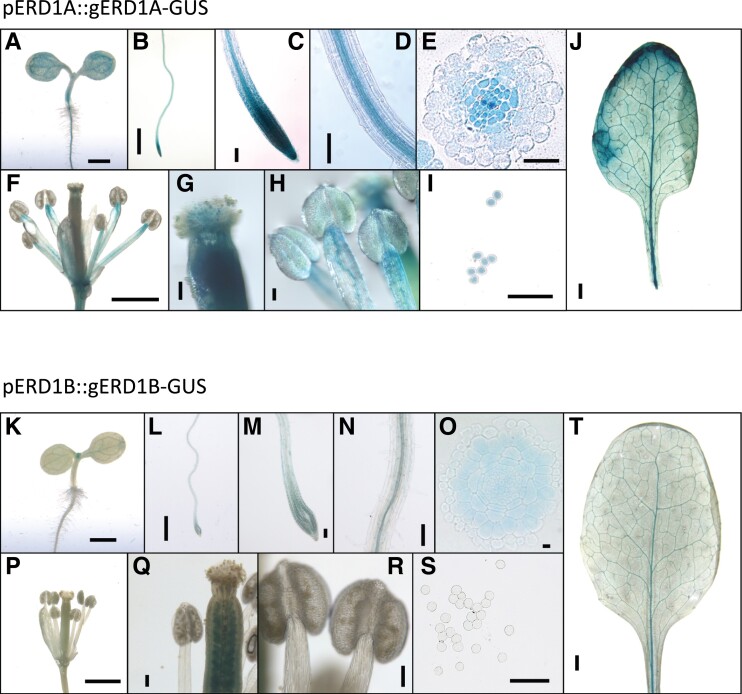
*ERD1A* and *ERD1B* are widely expressed in various tissues. The β-glucuronidase (GUS) staining profile for the *erd1a* mutant complemented by the *pERD1A::ERD1A-GUS* construct and incubated for 6 h in X-GlcA **A)–J)** and for WT plants transformed with the *pERD1B::ERD1B-GUS* construct and incubated for 16 h in X-GlcA **K)–T)**. The GUS profile is shown for 3-d-old seedlings **A)**, **K)** and 7-d-old roots **B)**–**E)**, **L)**-**O)**; flowers **F)**, **P)**; pistils **G)**, **Q)**; stamens **H)**, **R)**; pollen grains **I)**, **S)**; and mature leaves **J)**, **T)**. Scale bars, 1 mm **A)**, **B)**, **F)**, **J)**, **K)**, **L)**, **P)**, and **T)**; 100 *µ*m **C)**, **D)**, **G)**, **H)**, **I)**, **M)**, **N)**, **Q)**, **R)** and **S)**; and 10 *µ*m **E)** and **O)**.

We examined the *ERD1A* and *ERD1B* expression patterns in transgenic plants transformed with the β-glucuronidase (GUS) reporter fused in-frame at the 3′ end of each gene using genomic DNA fragments that included 1.2 kb of the promoter region. The *pERD1A::gERD1A-GUS* construct, which complemented the *erd1a* mutant (Supplemental Fig. S4), showed a broad GUS expression pattern after 6 h incubation with the substrate 5-bromo-4-chloro-3-indolyl glucuronic acid (X-GlcA), including in the root meristem and vascular cylinder, the rosette leaf vasculature and lamina, and the stamen filament, pistil, and pollen grains (Fig. 2, A–J). In contrast, plants transformed with the *pERD1B::gERD1B-GUS* construct had a similar but much weaker staining profile after 16 h of X-GlcA incubation in the cotyledon, root, rosette leaf, and pistil, but failed to show staining in the stamen and pollen (Fig. 2, K–T). Analysis of published RNA-seq data from Arabidopsis roots and leaves revealed that *ERD1A* expression was 3.8- and 2.9-fold higher than *ERD1B* in roots and leaves, respectively (Deforges et al. 2019; Supplemental Table S1). The higher level of *ERD1A* expression relative to *ERD1B* in both roots and leaves was further confirmed by reverse transcription quantitative PCR (RT-qPCR) (Fig. 1N). In summary, although *ERD1A* and *ERD1B* expression profiles largely overlap in numerous tissues, except in the pollen, *ERD1A* showed a consistently higher level of expression than *ERD1B*.

Considering the relatively weak expression level of *ERD1B* and the amino acid homology between ERD1A and ERD1B, we aimed to determine if ERD1B could have a similar function as ERD1A and could complement the *erd1a* mutant if its level of expression was increased. This was analyzed by generating homozygous *erd1a* mutants expressing the *ERD1A-GFP* and *ERD1B-GFP* constructs under the control of either the *ERD1A* or CaMV35S promoter. Following analysis of approximately 10 independent lines for each construct, 2 lines expressing the highest level of each transgene were analyzed and compared. Analysis of transgene expression by RT-qPCR showed that maximal expression of the *ERD1B* transgene under the *ERD1A* and *CaMV35S* promoter was substantially lower than the homologous constructs using the *ERD1A* gene, indicating a potential role for posttranscriptional regulation in the level of *ERD1A* and *ERD1B* expression (Fig. 1O). While partial complementation of *erd1a* was obtained by the relatively weak expression of *ERD1B* using the *pERD1A::ERD1B-GFP* construct, full *erd1a* complementation was obtained via the stronger expression of *ERD1B-GFP* using the CaMV35S promoter (Fig. 1, O and P). These results indicate that *ERD1B-GFP* can complement the *erd1a* mutant when expressed at levels similar to those of *pERD1A::ERD1A-GFP* lines and, thus, that *ERD1A* and *ERD1B* have similar functions. The lack of phenotype of the *erd1b* mutant is likely due to the weaker expression of *ERD1B* relative to *ERD1A*.

We tested whether ERD1A could complement PHT4:6 activity. Transforming the *35S::ERD1A-GFP* construct into the *pht4:6* mutant failed to complement its rosette growth phenotype. Although the *35S::PHT4:6-GFP* construct complemented the *pht4:6* mutant, it failed to complement the *erd1a* mutant phenotypes (Supplemental Fig. S5). This indicates that ERD1A and PHT4:6 have distinct functions in plants.

**Figure 3.**
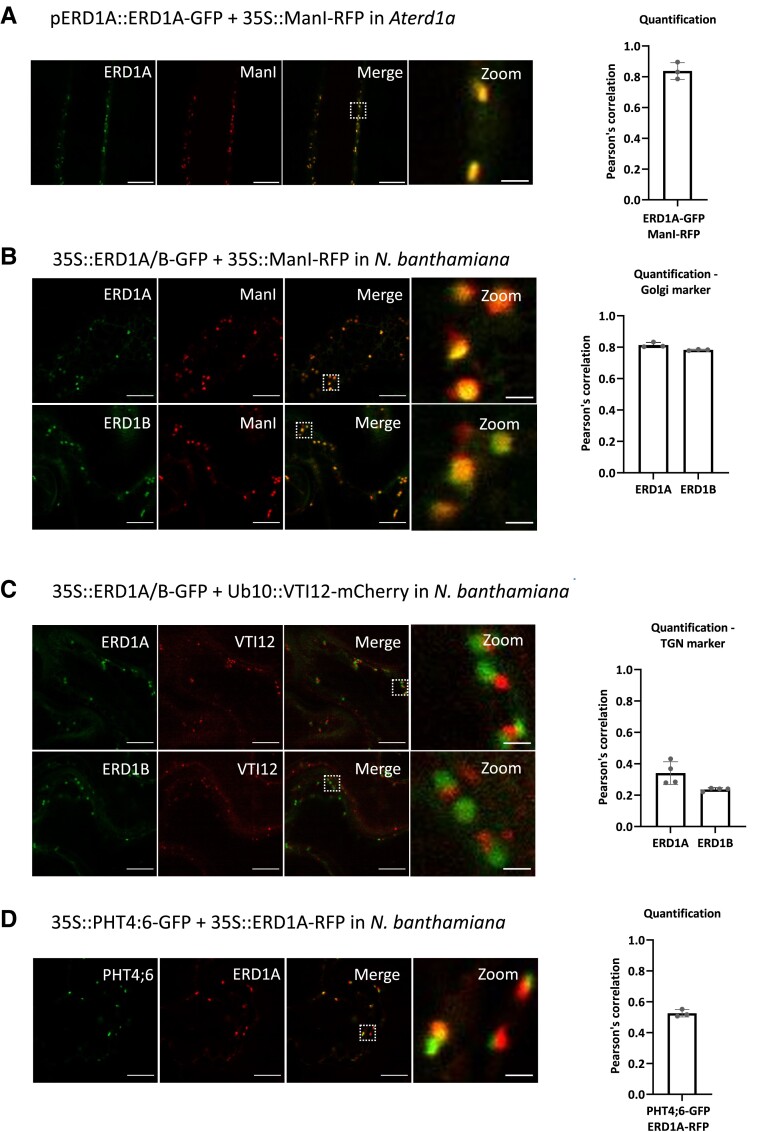
Subcellular localization of ERD1A and ERD1B. **A)** Fluorescent signals from the root of the transgenic line co-expressing ERD1A-GFP with the *ci*s-Golgi marker ManI-RFP in the *erd1a* mutant. Epidermal cells from *N. benthamiana* leaf discs transiently expressing ERD1A-GFP or ERD1B-GFP with ManI-RFP **B)** and the *trans*-Golgi network (TGN)-early endosome marker VTI12-mCherry **C)**. **D)** Co-expression of PHT4;6-GFP and ERD1A-RFP in an *N. benthamiana* leaf. The white-dashed square in the merged pictures indicates the zoomed-in area. The Pearson's correlation indicating the colocalization level for each combination is shown on the right. Scale bars for the 3 left panels = 10 *µ*m, while scale bar for the right panel representing a zoomed-in area = 1 *µ*m. Error bars, ±sd, *n* ≥ 3.

We examined the subcellular localization of ERD1A and ERD1B using GFP fusions. Expressing the *cis*-Golgi marker ManI fused to red fluorescent protein (ManI-RFP) in *erd1a* complemented with *pERD1A::ERD1A-GFP* resulted in a high level of RFP and GFP co-localization in the root (Fig. 3A). We observed similar co-localization between ManI-RFP and ERD1A-GFP or ERD1B-GFP in transiently transformed *Nicotiana benthamiana* leaves (Fig. 3B), but a poor overlap between ERD1A-GFP or ERD1B-GFP and the *trans*-Golgi network (TGN)/early endosome marker VTI12-mCherry (Fig. 3C). Furthermore, we observed strong co-localization when ERD1A-RFP and ERD1B-GFP were co-expressed (Supplemental Fig. S6). PHT4:6 localizes to the *trans*-Golgi (Hassler et al. 2012), and we observed a low level of co-localization between PHT4:6-GFP and ERD1A-RFP in *N. benthamiana* leaves (Fig. 3D). In summary, ERD1A and ERD1B localize in the *cis*-Golgi, while PHT4;6 localizes in the *trans*-Golgi.

### Arabidopsis *ERD1A* and *ERD1B* complement the yeast *erd1* mutant's defect in Pi homeostasis

**Figure 4.**
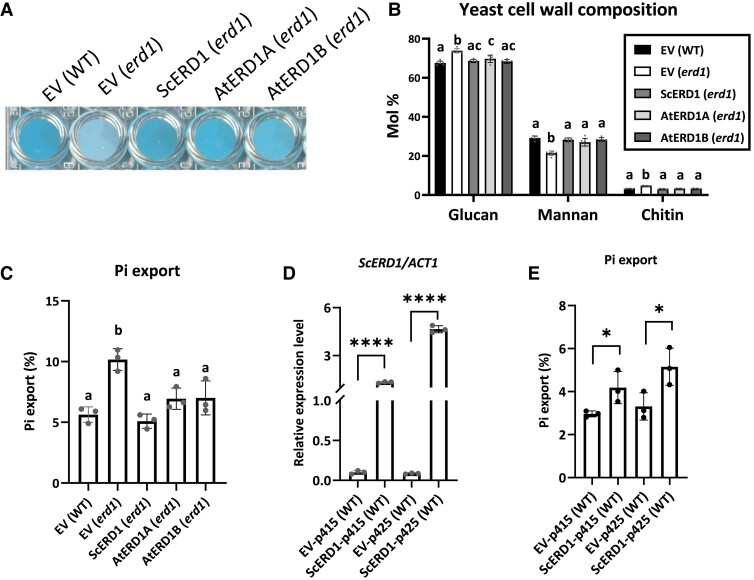
*ERD1A* and *ERD1B* complemented the yeast *erd1* phenotypes. Native *S. cerevisiae ERD1* and codon-optimized Arabidopsis *ERD1A* and *ERD1B* were expressed under the native Sc*ERD1* promoter. The empty vector (EV) was transformed into the WT and the *erd1* mutant as controls. *ScERD1*, *AtERD1A*, and *AtERD1B* complemented the *erd1* Alcian blue staining phenotype **A)**, cell wall composition **B)**, and increased Pi export after 4 h of incubation **C)**. For **B)** and **C)**, different letters indicate significant differences by 1-way ANOVA and Tukey's multiple comparisons test, Error bars, ±Sd, *n* ≥ 3. *ScERD1* expression level measured by RT-qPCR **D)** and Pi export after 6 h incubation **E)** of WT *S. cerevisiae* cells transformed with *ScERD1* under the control of the strong *GPD* promoter present in the low- and high-copy vectors p415 and p425, relative to the EV controls. Values in **D)** are relative to *ACT2*. For **D** and **E**, Student's *t*-test, *P < 0.05; ****P < 0.0001, Error bars, ±Sd, *n* ≥ 3.

Next, we assessed the ability of ERD1A and ERD1B to complement yeast *erd1* mutant phenotypes. We expressed codon-optimized *ERD1A* and *ERD1B* as well as native *ScERD1* under the control of the *ScERD1* promoter in the yeast *erd1* mutant. *ERD1A*, *ERD1B*, and *ScERD*1 complemented the reduced cell wall Alcian blue staining observed in the empty vector-transformed *erd1* mutant (Fig. 4A). Furthermore, the yeast *erd1* cell wall had reduced mannan contents relative to the wild type (WT), but *ERD1A*, *ERD1B*, and *ScERD1* complemented this phenotype in transgenic yeast (Fig. 4B). However, *ERD1A* and *ERD1B* did not complement the *erd1* mutant phenotype of increased export of the ER luminal protein BiP to the apoplastic space (Supplemental Fig. S7).

The yeast *erd1* mutant has previously been shown to transport excessive amounts of Pi from the Golgi to the apoplastic space via exocytosis (Snyder et al. 2017). Consistent with these data, expression of *ERD1A*, *ERD1B*, and *ScERD*1 under the control of the *ScERD1* promoter restored apoplastic Pi export to WT levels (Fig. 4C). Remarkably, *ScERD1* overexpression in WT yeast using the strong *GPD* promoter, present in the low-copy centromeric plasmid p415 or the high-copy 2 *µ* plasmid p425, also resulted in increased apoplastic Pi export relative to the empty vector control (Fig. 4, D and E).

### *ERD1A* inactivation and overexpression in plants are associated with Pi export to the apoplast

**Figure 5.**
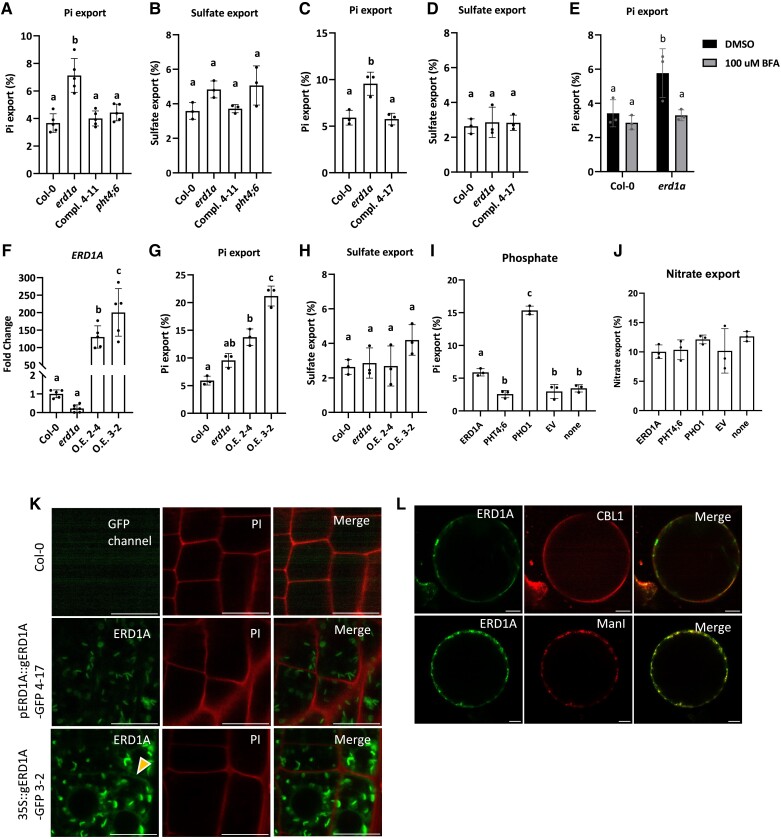
Pi export in the *erd1a* mutant and *ERD1A* overexpression lines. ^33^Pi export to the apoplast in 7-d-old seedlings of Col-0 **A**, **C**, **E**, **G)**, the *erd1a* mutant **A**, **C**, **E**, **G)**, *erd1a* complemented by *pERD1A::ERD1A-GFP* in lines 4–11 **A)** and 4–17 **C)**, the *pht4;6* mutant **A)**, and *erd1a* overexpressing *ERD1A* from the *p35S::ERD1A-GFP* construct in lines O.E. 2–4 and O.E. 3–2 **G)**. For the same lines, ^35^SO_4_ export was tested as controls **B**, **D**, **H)**. The effect of adding 100 *µ* M BFA on ^33^Pi export was tested in Col-0 and *erd1a***E)**. Level of *ERD1A* transcript in the overexpression lines O.E. 2–4 and O.E. 3–2 compared to Col-0 and the *erd1a* mutant **F)**. ^33^Pi export to the apoplast in *N. benthamiana* leaf discs overexpressing *ERD1A-GFP*, *PHT4;6-GFP*, or *PHO1-GFP* was compared to the empty vector (EV) control and un-infiltrated plants after a 6-h incubation **I)**. The nitrate export level was tested as a control in the same samples **J)**. For **A)** to **J)**, different letters indicate significant differences by 1-way ANOVA and Tukey's multiple comparisons test. Error bars, ±Sd, *n* ≥ 3. **K)** Seedlings of Col-0, *erd1a* line 4–17 complemented with *pERD1A::ERD1A-GFP*, and line O.E. 3–2 overexpressing *ERD1A* from the *p35S::ERD1A-GFP* construct were stained with propidium iodide (PI) to delineate the plasma membrane. The same confocal laser settings were applied in all images. The yellow arrowhead indicates the partial colocalization of the ERD1A-GFP signal with the PI stain. **L)** Transiently overexpressed ERD1A-GFP in *N. benthamiana* protoplasts showed partial colocalization with CBL1-OFP, a plasma membrane marker, and with ManI-RFP, a *cis*-Golgi marker. Scale bars for K and L, 10 *µ*m.

We assessed the role of Arabidopsis ERD1A in Pi homeostasis in the *erd1a* mutant. Similar to the yeast *erd1* mutant, we observed 2-fold higher apoplastic Pi export activity in the Arabidopsis *erd1a* mutant compared to the Col-0 WT and the 2 *pERD1A::ERD1A-GFP*-complemented *erd1a* lines (complemented lines 4–11 and 4–17) (Fig. 5, A and C). Pi export to the apoplast was unaffected in the Arabidopsis *pht4;6* mutant and was similar to the Col-0 WT control. In contrast to Pi export, sulfate export to the apoplast was similar in all lines tested, indicating the specificity of ERD1A for Pi export (Fig. 5, B and D).

In the yeast *erd1* mutant, increased apoplastic Pi export was found to be dependent on exocytosis (Snyder et al. 2017). Similarly, the increased Pi export observed in the Arabidopsis *erd1a* mutant was abolished by treatment with Brefeldin A (BFA) (Fig. 5E). BFA inhibits exocytosis as well as other aspects of the Golgi vesicular trafficking, including retrograde transport, in part through interfering with Guanine nucleotide exchange factors (GEF) and ADP-ribosylation factor 1 (Arf1) (Niu et al. 2005; Huang and Zhang 2020). These results suggest that the increased Pi export observed in the Arabidopsis *erd1a* mutant is dependent on components of vesicular trafficking implicating the Golgi.

Next, we examined the effect of *ERD1A* overexpression on Pi export in 1-wk-old seedlings of 2 independent *erd1a* lines transformed with *35S::ERD1A-GFP*; these overexpression (O.E) lines showed 100- to 200-fold O.E of *ERD1A* relative to Col-0 (Fig. 5F). The O.E lines had a 2-fold (line O.E 2–4) and 3-fold (line O.E 3–2) increase in Pi export relative to Col-0, while sulfate export remained unchanged (Fig. 5, G and H). Next, we examined Pi export dynamics via transient expression in *N. benthamiana* leaves. Transient expression of *PHT4;6-GFP* did not alter Pi export relative to the empty vector (EV) control or un-infiltrated leaves, but transient expression of *ERD1A-GFP* led to a 3-fold increase in Pi export, and transient expression of *PHO1-GFP* led to a 7-fold increase in Pi export relative to the EV control (Fig. 5I). However, transient expression of *ERD1A-GFP*, *PHT4;6-GFP*, and *PHO1-GFP* did not alter nitrate export (Fig. 5J).

The increase in Pi export activity observed in the Arabidopsis *erd1a* transgenic lines overexpressing *ERD1A-GFP* from the CaMV35S promoter but not in *erd1a* lines expressing *ERD1A-GFP* from the native *ERD1A* promoter suggested that overexpression results in mislocalization of ERD1A. Indeed, a close examination of GFP localization in the *35S::ERD1A-GFP*-overexpression line 3–2 revealed a weak fluorescent signal at the plasma membrane in addition to the previously observed signal in the Golgi (Fig. 5K). In contrast, the GFP signal in line 4–17 complemented with *pERD1A::ERD1A-GFP* appeared to be restricted to the Golgi. Similarly, protoplasts from a section of the *N. benthamiana* leaf infiltrated with the *35S::ERD1A-GFP* construct showed a partial GFP co-localization with the plasma membrane marker Calcineurin B-like calcium sensor 1-orange fluorescent protein (CBL1-OFP) in addition to the co-localization with the Golgi marker ManI-RFP (Fig. 5L).

### The *erd1a* mutant has altered cell wall composition as well as reduced hypocotyl and primary root elongation

**Figure 6.**
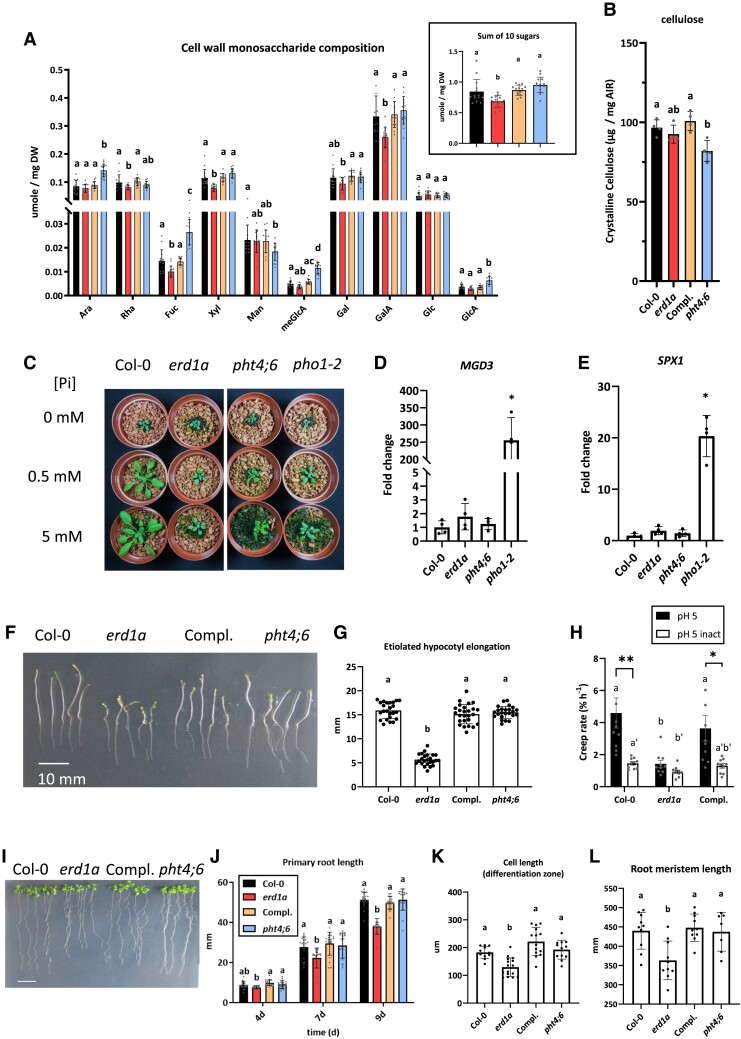
Impact of the *erd1a* mutation on cell wall composition, hypocotyl elongation, and root growth. The cell wall monosaccharide composition **A)** and crystalline cellulose content **B)** from 4-wk-old mature leaves of Col-0, *erd1a*, *erd1a* complemented with the *pERD1A::ERD1A-GFP* construct (Compl.), and *pht4;6.* Monosaccharides analyzed are arabinose (Ara), rhamnose (Rha), fucose (Fuc), xylose (Xyl), mannose (Man), methylglucuronic acid (meGlcA), galactose (Gal), galacturonic acid (GalA), glucose (Glc), and glucuronic acid (GlcA). For **A)** and **B)**, different letters indicate significant differences by 1-way ANOVA and Tukey's multiple comparisons test. Error bars, ±Sd, *n* ≥ 14. Inset in **A)** shows the sum of all 10 sugars for the 4 genotypes. **C)** Four-week-old plants grown in soil-free clay substrate with different Pi concentrations. RT-qPCR analysis of Pi starvation-responsive markers *MGD3***D)** and *SPX1***E)** in 4-wk-old soil-grown plants. Expression levels show the fold change relative to Col-0, which was set to 1. For **D)** and **E)**, Student's *t*-test to Col-0, error bars, ±Sd, *n* ≥ 4 (*P < 0.05). **F)** and **G)** Etiolated hypocotyls were measured 6 d after incubation in the dark. Scale bar, 10 mm. Different letters indicate significant differences by 1-way ANOVA and Tukey's multiple comparisons test. Error bars, ±Sd, *n* ≥ 24. **H)** Differences in creep rates of equivalent length (7 mm) dark-grown hypocotyls of Col-0, *erd1a*, and *erd1a* complemented with the *pERD1A::ERD1A-GFP* construct (Compl.) under a 1-g load. To attain the equivalent length, hypocotyls of Col-0 and Compl lines were grown for 3 d, while *erd1a* hypocotyls were grown for 7 d. Abbreviations: pH 5, frozen/thawed hypocotyls extended at pH 5; pH 5 inact, frozen/thawed heat-inactivated hypocotyls extended at pH 5; asterisks indicate significant differences (*P < 0.05; **P < 0.01, Student's 2-tailed *t*-test) for a given line between pH 5 and pH 5 inact; different letters a, b, and c indicate significant differences (*P* < 0.05, Bonferroni-corrected Student's *t*-test) between different lines at pH 5; the letters a′ and b′ indicate significant differences (*P* < 0.05, Bonferroni-corrected Student's *t*-test) between different lines at pH 5 inact. Error bars, ±Se, *n* ≥ 10. **I)–L)** Growth of primary roots on MS medium for Col-0, *erd1a*, *erd1a* complemented with the *pERD1A::ERD1A-GFP* construct (Compl.), and *pht4;6* (I, scale bar, 10 mm). **J)** Root length was measured 4, 7, and 9 d after germination (DAG). Roots of 9 DAG seedlings were analyzed for cell length in the differentiation zone **K)** and the length of the meristem zone **L)**. For **J)** to **L)**, different letters indicate significant differences by 1-way ANOVA and Tukey's multiple comparisons test. Error bars, ±Sd, *n* ≥ 18.

Since Pi flux in the Golgi is associated with NDP-sugar import, we analyzed the cell wall monosaccharide composition in rosette leaves. The *erd1a* and *pht4;6* mutants showed limited but distinctive changes in cell wall monosaccharide composition (Fig. 6A). Compared to Col-0, the *erd1a* mutant cell wall had small decreased content of rhamnose, fucose, xylose, and galacturonic acids, whereas the *pht4;6* mutant had small increased content of arabinose, fucose, methylglucuronic acid, and glucuronic acids, and decreased mannose content. When we calculated the sum of the 10 sugars analyzed per dry leaf weight, the *erd1a* mutant had a lower value for this parameter, which remained unchanged in the *pht4;6* mutant (Fig. 6A insert). We did not observe a significant change in the crystalline cellulose content in *erd1a* relative to the WT but did observe a small decrease in this parameter in *pht4;6* (Fig. 6B). Furthermore, we observed only minor changes in the nucleotide sugar content in *erd1a* leaves compared with Col-0, with a small increase in UDP-GalA and UDP-GlcNAc as well as a small decrease in UDP-Gal (Supplemental Fig. S8).

We further examined the link between ERD1A and Pi homeostasis by growing *erd1a*, *pht4;6*, and *pho1* mutants in a clay-based substrate fertilized with half-strength Murashige and Skoog (MS) with no Pi, 0.5 mM Pi, or 5 mM Pi. Increasing the Pi content of the medium did not rescue the reduced shoot and root growth phenotypes of *erd1a*, and the effect was also limited in *pht4;6* and *pho1* (Fig. 6C, Supplemental Fig. S9, A and B). Despite the strong reduction in root and shoot growth, the *erd1a* mutant had a similar shoot and root Pi content as WT plants under all tested conditions (Supplemental Fig. S9, C and D). Analysis of the vacuolar Pi content of plants grown in soil by in vivo nuclear magnetic resonance spectroscopy also revealed no significant differences between WT and *erd1a* plants (Supplemental Fig. S9E). While 2 phosphate starvation-regulated genes, *MGD3* and *SPX1*, were strongly upregulated in the Pi-deficient shoots of soil-grown *pho1–2*, we did not observe a significant difference in their expression in *erd1a* and *pht4;6* compared to the WT (Fig. 6, D and E). This indicates that the *erd1a* and *pht4;6* mutant phenotypes are not associated with a strong perturbation in whole-plant Pi homeostasis that would lead to phosphate starvation responses.

In addition to the strong reduction of rosette and root growth of *erd1a* grown under phototrophic conditions, hypocotyl growth of seedlings grown in the dark on MS media containing 1% sucrose was also strongly reduced in *erd1a* but not in *pht4;6* (Fig. 6, F and G). When compared at the equivalent length of 7 mm, the creep rate (an estimate of cell wall extensibility) of *erd1a* hypocotyls was one-third than that of Col-0 and *erd1a* complemented with *pERD1A::ERD1A-GFP* at pH 5, the condition when expansins, the most potent cell wall-loosening proteins, are active. This difference was diminished after heat inactivation to eliminate the activities of endogenous cell wall-loosening proteins (Fig. 6H).

When grown in an agar-supplemented medium containing 1% sucrose, the *erd1a* mutant had shorter primary roots compared to the WT and *erd1a* complemented with *pERD1A::ERD1A-GFP*, while *pht4;6* roots were similar to WT roots (Fig. 6, I and J). The reduction in *erd1a* primary root growth was associated with reduced cell size in the differentiation zone and reduced size of the root meristematic zone, indicating an effect on meristematic cell division and cell elongation in the root (Fig. 6, K and L).

### *ERD1A* affects pollen fitness, and absence of *ERD1A* and *ERD1B* abolishes male but not female gamete transmission

A segregating population of 120 seedlings from the self-fertilization of a heterozygous *ERD1A*/*erd1a* parent had 9 *erd1a*/*erd1a* homozygous mutant plants, significantly deviating from the expected 1:3 ratio (χ^2^ test, *P* < 0.01). We did not observe embryo lethality in developing seeds from siliques of *ERD1A*/*erd1a* plants (Supplemental Fig. S10). Furthermore, the progeny from reciprocal crosses between *ERD1A*/*erd1a* and *ERD1A*/*ERD1A* parents had reduced transmission of the *erd1*a allele only through the pollen, and this phenotype was complemented by the *pERD1A::ERD1A-GFP* construct (Table 1).

**Table 1.** Analysis of the transmission of the *erd1a* allele in the male and female gametes

	Parental genotype		Progeny genotype at the *ERD1A* locus		*P*-value (Fisher's exact test)
	Pollen donor	Ovule donor	*ERD1A*/*erd1a*	*ERD1A*/*ERD1A*	
(a)	*ERD1A*/*erd1a*	*ERD1A*/*ERD1A*	10 (23%)	33 (77%)	0.014
	*ERD1A*/*ERD1A*	*ERD1A*/*erd1a*	18 (49%)	17 (51%)	1
	*ERD1A*/*erd1a*	*ERD1A*/*ERD1A*	52 (58%)	37 (42%)	0.32
	*ERD1A-GFP*/*ERD1A-GFP*				
(b)	*ERD1A*/*erd1a*	*ERD1A*/*ERD1A*	0 (0%)	36 (100%)	<0.001
	*erd1b*/*erd1b*	*erd1b*/*erd1b*			
	*ERD1A*/*ERD1A*	*ERD1A*/*erd1a*	15 (43%)	20 (57%)	0.40
	*erd1b*/*erd1b*	*erd1b*/*erd1b*			

**Figure 7.**
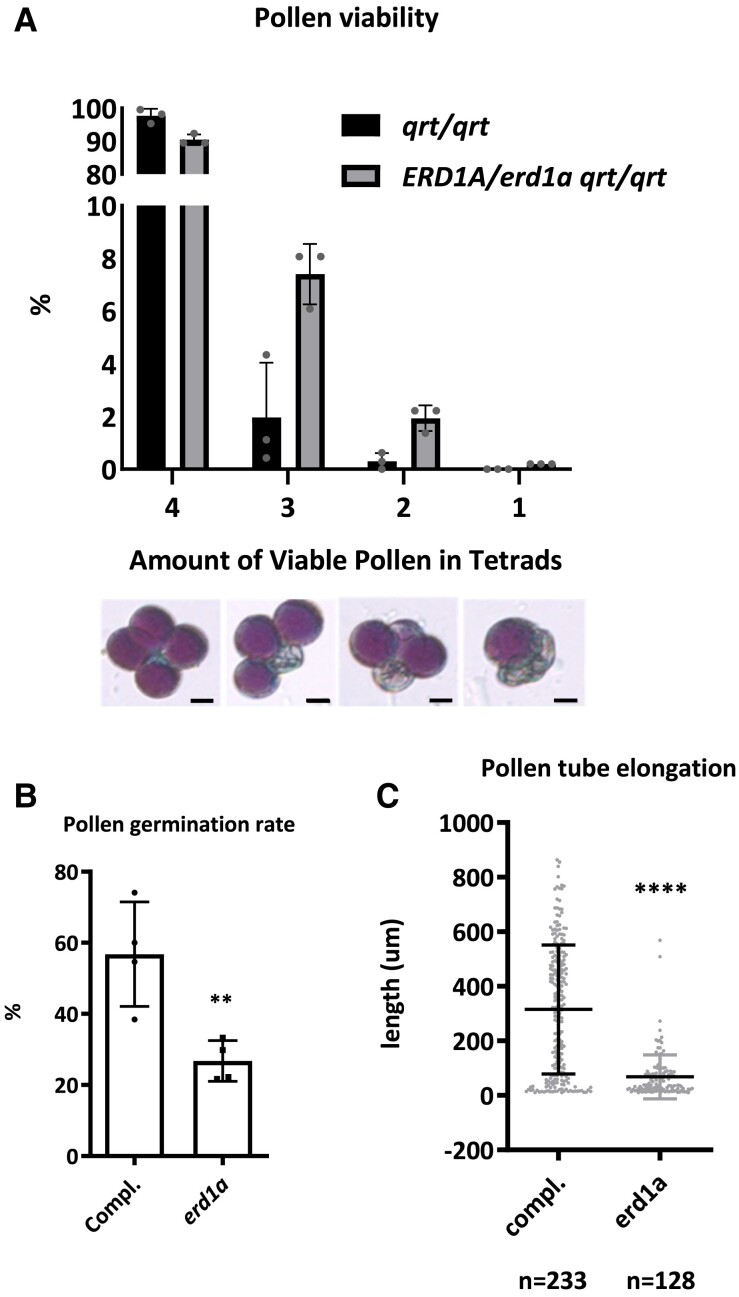
Analysis of pollen viability, germination, and elongation. **A)** The percentage of viable pollen in each tetrad from *qrt*/*qrt* and *ERD1A*/*erd1a qrt*/*qrt* plants. Three independent experiments were performed, and more than 200 tetrads were counted for each genotype in each experiment. Statistics: Chi-square, error bars ± Sd, *n* = 300 pooled into 3 sets. Scale bars in the lower panel represent 10 *µ*m. **B)** Pollen was collected for in vitro germination from the *erd1a* mutant heterozygous for the complementing construct *pERD1A::ERD1A-GFP* (*ERD1A-GFP*/- *erd1a*/*erd1a*). After 5 h of incubation in the germination solution, the elongated pollen tubes were checked under a confocal microscope to distinguish between pollen expressing *pERD1A::ERD1A-GFP* (GFP positive) and *erd1a* pollen without the construct (GFP negative). The germination rate **B)** and pollen tube elongation **C)** were then measured. Pollen was determined as germinated when the length of the pollen tube exceeded the pollen radius. For **B)** and **C)**, Student's *t*-test, **P < 0.01; ****P < 0.0001, Error bars, ±Sd. For **B)**, *n* = 100 pollen pooled into 4 sets. For **C)**, n ≥ 128.

Based on Alexander staining, we did not observe a significant difference in the viability of pollen isolated from *ERD1A*/*erd1a qrt*/*qrt* compared to the *qrt*/*qrt* control (Fig. 7A). To analyze the pollen germination rate and pollen tube elongation, we harvested pollen from homozygous *erd1a*/*erd1a* plants that were heterozygous for the complementing construct *pERD1A::ERD1A-GFP*, which is expressed in pollen grains. The germination rate and tube elongation of GFP-negative pollen grains (*erd1a*) were reduced compared to GFP-positive pollen grains (*erd1a* complemented with *pERD1A::ERD1A-GFP*) (Fig. 7, B and C). These results reveal that, although *erd1a* pollen produced from heterozygous *ERD1A*/*erd1a* plants is viable, it has reduced fitness compared to WT pollen due to reduced pollen germination and elongation, explaining the skewed progeny segregation ratio from *ERD1A*/*erd1a* parents.

We did not recover a double mutant (*erd1a*/*erd1a erd1b*/*erd1b*) from our analysis of over 300 progenies from self-fertilized *ERD1A*/*erd1a erd1b*/*erd1b* plants, and there was no evidence of embryo lethality in developing siliques (Supplemental Fig. S10), indicating a likely defect in the *erd1a erd1b* gametophytes. Therefore, we analyzed progeny from reciprocal crosses between *ERD1A*/*erd1a erd1b*/*erd1b* and *ERD1A*/*ERD1A erd1b*/*erd1b* plants to assess the requirement of *ERD1A* in the *erd1b*/*erd1b* background in male and female gametophytes for gamete transmission. Transmission of the *erd1a erd1b* and *ERD1A erd1b* alleles via the female gametophyte was equal, but we only detected transmission of the *ERD1A erd1b* alleles via pollen (Table 1). Thus, while the absence of ERD1A and ERD1B was not deleterious to the female gametophyte, the presence of ERD1A or ERD1B is necessary for pollen development and/or viability, although the absence of only ERD1A leads to reduced pollen fitness.

## Discussion

The EXS domain is found in 2 groups of eukaryotic proteins (Wang et al. 2008). The first group includes PHO1 homologs, which harbor EXS and SPX domains, with SPX involved in inositol pyrophosphate binding (Wild et al. 2016; Jung et al. 2018). Plants, fungi, and animals contain PHO1 orthologs; plant PHO1 and its mammalian ortholog XPR1 export Pi to the apoplastic space (Arpat et al. 2012; Giovannini et al. 2013; Wege and Poirier 2014). The structure–function analysis of PHO1 revealed that its EXS domain is essential for Pi export activity (Wege et al. 2016). The second group of proteins possessing the EXS domain includes ERD1. Although fungi and a broad spectrum of plants, ranging from unicellular algae to bryophytes and vascular plants, contain ERD1 putative orthologs, none are present in mammals (human and mouse), insects (*Drosophila melanogaster*), and nematodes (*Caenorhabditis elegans*), indicating that the ERD1 was lost in the metazoan lineage.

Gene expression profiles using the GUS reporter as well as protein subcellular localization using fluorescent protein fusions indicated that *ERD1A* and *ERD1B* are broadly expressed *cis*-Golgi proteins. Proteomics of isolated Golgi from Arabidopsis confirmed the presence of ERD1A in this organelle (Parsons et al. 2012), and the *S. cerevisiae* ERD1 ortholog resides in *cis*-Golgi (Miyasaka et al. 2020; Sardana et al. 2021). Furthermore, the complementation of the *erd1a* mutant by overexpression of *ERD1B* using the *CaMV35S* promoter revealed that ERD1A and ERD1B proteins have similar functions. Characterization of Arabidopsis ERD1A and ERD1B as Golgi Pi exporters is supported by several lines of evidence. First, expression of either *ERD1A* or *ERD1B* using the *S. cerevisiae ERD1* promoter complemented the defective Golgi Pi export (Pi loss to the external media via exocytosis) in the *S. cerevisiae erd1* mutant (Snyder et al. 2017). We observed a similar phenotype of excessive loss of Pi to the apoplast in the Arabidopsis *erd1a* mutant; this phenotype was also dependent on BFA, indicating the participation of Golgi-associated vesicular transport processes such as exocytosis. Second, *ERD1A-GFP* overexpression with the CaMV35S promoter in plants led to Pi export to the apoplast, while sulfate and nitrate export was unaffected. We also observed Pi export to the apoplast via transient *ERD1A-GFP* expression in *N. benthamiana* and stable *ScERD1* overexpression in yeast.

In Arabidopsis and *N. benthamiana* overexpressing *ERD1A-GFP*, we observed a small proportion of the corresponding fusion protein in the plasma membrane in addition to the major proportion in the Golgi. Excess ERD1A-GFP accumulation may overwhelm the Golgi protein retrieval system in charge of maintaining resident Golgi proteins within the organelle, resulting in some ERD1A-GFP associating with exosomes that fuse with the plasma membrane. Thus, the increased apoplastic Pi export activity triggered by *ERD1A-GFP* overexpression would be mediated by a sub-population of ERD1A-GFP mislocated to the plasma membrane. In contrast, the increased apoplastic Pi export observed in both the yeast and plant *erd1* mutants is likely a result of the reduction of Pi export out of the Golgi, which would lead to excess Pi content in Golgi-derived vesicles. The fusion of Golgi-derived vesicles with high Pi content to the plasma membrane in the process of exocytosis would result in excessive apoplastic Pi export. For both the *erd1a* mutant and *ERD1A-GFP*-overexpression lines, the specificity observed for Pi export but not for nitrate or sulfate export indicates that the Pi export activity is mediated by a transporter and is not a consequence of membrane leakage.

The Pi content in roots and shoots and the vacuolar Pi concentration remained unchanged in the *erd1a* mutant relative to the WT. There was also no activation of phosphate starvation-responsive genes. Furthermore, increasing the external Pi supply did not rescue the *erd1a* mutant phenotype. Together, these results indicate that the aberrant Golgi Pi homeostasis observed in the *erd1a* mutant did not affect the whole-plant Pi content.

*ERD1* disruption alters the cell wall composition in yeast and plants. The cell wall of the yeast *erd1* mutant had reduced amounts of mannan, suggesting that the lack of ERD1 affects mannan elongation requiring GDP-mannose and the action of Golgi-localized Man-transferases (Orlean 2012). The *erd1a* mutant cell wall had a small but significant reduction in several monosaccharides, including rhamnose, fucose, xylose, galactose, and galacturonic acid, which reduced the sum of all 10 monosaccharides measured in the cell wall. This phenotype indicates that loss of *ERD1A* broadly affects the import of a variety of UDP-sugars into the Golgi, which may in turn affect the biosynthesis of several cell wall hemicellulose and pectin components and that of other glycosylated molecules, including lipids. Surprisingly, there was a minimal change in total (cytosolic and organellar) NDP-sugars, although this may not represent NDP-sugar levels in the Golgi. Limited changes in cell wall monosaccharide composition can be associated with strong developmental defects and are likely to contribute to the overall growth defect of *erd1a* rosettes and to the reduction in hypocotyl growth and cell wall extensibility, root elongation, pollen germination, and pollen tube elongation. For example, the null Arabidopsis *mur3-3* mutant, which is deficient in galactosyltransferase activity and contains cell walls with lower amounts of galactosylated xyloglucans, has strongly reduced rosette growth and shorter hypocotyls with reduced cell wall extensibility (Madson et al. 2003; Pena et al. 2004; Tamura et al. 2005; Kong et al. 2015). Similarly, deficiency in cell wall rhamnogalacturonan-II dimerization reduces root cell elongation (Dumont et al. 2015), while perturbed homogalacturonan biosynthesis leads to defects in pollen germination, pollen tube elongation, and male fertility (Lund et al. 2020).

An increase in the Golgi Pi concentration resulting from defective Pi export may directly or indirectly affect numerous pathways involving glycosylation. For example, an in vitro assay using potato (*Solanum tuberosum*) apyrase revealed that the addition of 125 *µ* M Pi resulted in a 50% reduction in apyrase ATPase activity (Karamohamed and Guidotti 2001). Since NDP-sugar import into the Golgi is directly coupled to NMP export, inhibiting the conversion of NDP (liberated by glycosyltransferases) to NMP by Golgi apyrases would decrease the flux of NDP-sugars into the Golgi. The Arabidopsis genome contains 7 apyrases (APY1 to APY7), of which 5 localize in the Golgi (Chiu et al. 2015). Although it is currently unknown if any of these Golgi apyrases are inhibited by Pi, it is interesting to note that the Arabidopsis double-mutant *apy1 apy2* has reduced pollen germination, while the *apy6 apy7* mutant has defective pollen exine formation and reduced male fertility, supporting a role of apyrases in Golgi polysaccharide biosynthesis, including in the pollen (Steinebrunner et al. 2003; Yang et al. 2013).

Although *ERD1A* and *PHT4;6* are not homologs, both are implicated in Golgi Pi homeostasis. The failure to complement the *erd1a* and *pht4:6* mutants with the *35S::PHT4;6-GFP* and *35S::ERD1A-GFP* constructs, respectively, and the distinct *erd1a* and *pht4:6* phenotypes relating to cell wall monosaccharide composition, apoplastic Pi export, as well as root and hypocotyl growth suggest that *erd1a* and *pht4:6* have distinct functions. This may be related to their distinct sub-cellular localization, with ERD1A localized primarily in the *cis*-Golgi, while PHT4;6 is localized to the *trans*-Golgi. Protein and lipid glycosylation, as well as pectin and hemicellulose glycan biosynthesis, are highly dynamic processes, during which sugar addition, removal, and modification alter carbohydrate structures as they progress from the *cis*-Golgi to the *trans*-Golgi. Consequently, various glycosyltransferases, glycosidases, sugar epimerases, and NSTs are differentially localized and maintained in the various Golgi stacks (Stanley 2011; Fisher and Ungar 2016; Parsons et al. 2019). The Arabidopsis genome encodes more than 40 putative nucleotide-sugar transporters, and their *cis*- and *trans*-Golgi localization supports the existence of NDP-sugar and Pi transport across the various Golgi stacks (Ebert et al. 2018; Parsons et al. 2019). Thus, the perturbed Pi export in the *cis*-Golgi caused by the absence of ERD1A would impact enzymes and glycan biosynthesis differently than the perturbed Pi export caused by the absence of PHT4;6 from the *trans*-Golgi. The absence of an apoplastic Pi export phenotype for the *pht4;6* mutant is puzzling and perhaps suggests that the quantitative export of Pi from the *cis*-Golgi via ERD1A/B is higher than in the *trans*-Golgi via PHT4;6. Clearly, further experiments are needed to distinguish the relative contributions of ERD1A/B and PHT4;6 to Golgi Pi transport dynamics.

## Materials and methods

### Yeast growth, cloning, transformation, and biochemical analysis

The yeast *S. cerevisiae* strain BY4742 (MATα *his3Δ1 leu2Δ0 lys2Δ0 ura3Δ0*) and the *erd1* mutant (*YDR414c::kanMX4*) were obtained from EUROSCARF (http://www.euroscarf.de). Genes were amplified and inserted via In-Fusion or restriction enzymes into the p415-GPD, p425-GPD, or p423-GPD vector containing the *TDH3* promoter or their derivatives in which the *TDH*3 promoter was replaced with the *ScERD1* promoter (Mumberg et al. 1995). The plasmids were introduced into *S. cerevisiae* using the lithium acetate procedure before selection for growth at 28 °C on synthetic medium (SM) containing 6.7 g L^−1^ yeast nitrogen base without amino acids, 5 g L^−1^ ammonium sulfate, 20 g L^−1^ glucose, and 0.69 g L^−1^ of the appropriate amino acid dropout supplement (Formedium) (Gietz et al. 1992).

For Alcian blue staining, cells were washed once with 50 mM acetic acid, resuspended in 1% (w/v) Alcian blue in 50 mM acetic acid for 5 min, and washed twice with 50 mM acetic acid (Corbacho et al. 2004). Analysis of apoplastic BiP secretion was performed as previously described using immunoblotting with anti-KAR2 (BiP) antibodies.

For the Pi export assay, yeast cells were inoculated overnight in SM-His medium and diluted into fresh SM-His low-Pi (500 *µ* M Pi) medium supplemented with 0.5 *µ*Ci/mL ^33^Pi. After incubating overnight at 28 °C, cells reaching the exponential growth phase were washed with ice-cold SM-His low-Pi medium 4 times and resuspended in fresh SM-His low-Pi medium. At the indicated time points, the supernatant was taken by centrifugation at 15,000 × *g* for 1 min. The ratio of ^33^Pi measured in the supernatant relative to the total intracellular ^33^Pi at time 0 was considered the Pi export percentage. Radioactivity was measured using Ultima Gold scintillation liquid in the Perkin Elmer tri-carb 2800TF scintillation counter.

The composition of yeast cell wall polysaccharides was analyzed from a cell wall hydrolysate as previously described (François 2006). Briefly, mannan, chitin, β-(1,3)-glucan, and β-(1,6)-glucan in the purified cell walls were determined by acid hydrolysis, and the released sugar monomers (mannose, glucose, and *N*-acetylglucosamine) were quantified by high-performance anion-exchange chromatography with pulsed amperometric detection (Dallies et al. 1998) (Supplemental Fig. S11).

### Arabidopsis growth conditions, cloning, and transformation

All Arabidopsis (*Arabidopsis thaliana*) plants used in this study, including mutants and transgenic plants, were in the Columbia (Col-0) background. For in vitro experiments, plants were grown in half-strength Murashige and Skoog (MS) salts (Duchefa M0255) containing 1% (w/v) sucrose and 0.7% (w/v) agar. For the Pi-deficient medium, MS salts without Pi (Caisson Labs, MSP11) were used. Pi buffer pH 5.7 [93.5% (w/v) KH_2_PO_4_ and 6.5% (w/v) K_2_HPO_4_] was added to obtain different Pi concentrations. Plants were also grown in soil or in a clay-based substrate (Seramis) supplemented with half-strength MS with different Pi concentrations. Growth chamber conditions were 22 °C, 60% humidity, and a 16-h-light/8-h-dark photoperiod with 100 μE/m^2^ per second of white light.

*ERD1A*, *ERD1B*, and *PHT4:6* genomic sequences were amplified and inserted into the pENTR2B-Amp vector by In-Fusion technology before being cloned into the destination vector pMDC107 [green fluorescent protein (GFP) C-fusion], pMDC164 [β-glucuronidase (GUS) C-fusion], or pMDC83 (GFP C-fusion with the CaMV 35S promoter) (Curtis and Grossniklaus 2003) via recombination between the attL and attR sites (Invitrogen). The ManI-red fluorescent protein (RFP) (Golgi), VTI12-mCherry (*trans*-Golgi network), and CBL1-OFP (plasma membrane) markers were previously described (Nelson et al. 2007; Geldner et al. 2009; Batistič et al. 2010). The constructs were introduced into *Agrobacterium tumefaciens* pGV3101 and used for stable transformation in Arabidopsis by floral dipping (Clough and Bent 1998) or transient expression in *Nicotiana benthamiana* (Arpat et al. 2012).

### Staining and microscopy

β-Glucuronidase (GUS) staining was performed by reaction with the substrate 5-bromo-4-chloro-3-indolyl glucuronic acid (X-GlcA) as previously described (Stefanovic et al. 2007). For the root sections, the seedlings were stained and fixed in an agarose block, then cut with a microtome. Photos were taken with a Leica MZ16 or DM5500 microscope. All fluorescent images in Arabidopsis seedlings and *N. benthamiana* leaves for subcellular localization were taken with a Leica Stellaris or a Zeiss LSM700 confocal microscope. Quantification of co-localization was performed using the intensity correlation analysis method implemented in the ImageJ plug-in (http://www.macbiophotonics.ca/imagej/).

### *Nicotiana benthamiana* infiltration and Pi export assay

An overnight culture of *A. tumefaciens* was pelleted and resuspended in infiltration buffer [10 mM MES-KOH (pH 5.6), 10 mM MgCl_2_, and 150 *µ* M acetosyringone]. The leaves of 4- to 5-wk-old *N. benthamiana* plants were used for infiltration with diluted cultures of *A. tumefaciens*. After 3 d, the leaves were randomly cut into 1-cm-diameter discs and soaked in a buffer containing 5 mM glucose, 10 mM MES-KOH (pH 5.6), 1 mM KCl, 0.5 mM CaCl_2_, 0.5 mM MgSO_4_, and 0.01% (v/v) Triton X-100. Pi and nitrate released in the buffer were measured by the molybdate assay (Ames 1966) and nitrate reductase assay (Barthes et al. 1995), respectively.

### ^33^Pi and ^35^SO_4_ export in Arabidopsis seedlings

Seven-day-old seedlings were washed in a buffer containing half-strength MS without Pi and with 0.05% (w/v) MES (pH 5.6) and 1% (w/v) sucrose and then incubated with 4.5 *µ*Ci/mL ^33^Pi in the same buffer supplemented with 10 *µ* M Pi for 2 h at room temperature with shaking. The seedlings were washed in ice-cold water and buffer with 1 mM Pi and then incubated in the same buffer (1 mM Pi) for ^33^Pi export at room temperature with shaking. The medium was collected and seedlings were incubated at 55 °C for 1 h with 10% (w/v) SDS. Radioactivity in the media and seedlings was measured using Ultima Gold scintillation liquid and the Perkin Elmer tri-carb 2800TF scintillation counter.

In the ^35^SO_4_ export experiment, the same procedure was applied but using a buffer without sulfate for washing and buffer supplemented with 10 *µ* M MgSO_4_ and 4.5 *µ*Ci/mL ^35^SO_4_ for isotope labeling and 50 *µ* M for ^35^SO_4_ export.

### Etiolated hypocotyl biomechanics

Dark-grown Arabidopsis seedlings were frozen in liquid nitrogen. Some seedlings were heat-inactivated after thawing to eliminate the activity of endogenous cell wall-loosening/tightening enzymes. In vitro extension of frozen/thawed hypocotyls was measured with a custom-built constant load extensometer (Suslov and Verbelen 2006). A 3-mm-long hypocotyl segment was secured between clamps of the extensometer located 1.5 mm below cotyledons and preincubated in 20 mM sodium acetate buffer (pH 5.0) in the relaxed state for 2 min. Then, its time-dependent extension (creep) was measured in the same buffer under 1,000- or 2,000-mg loads for 15 min. The relative creep rate was calculated as previously described (Vandenbussche et al. 2011).

### Pollen viability, germination, and pollen tube elongation

Pollen viability was analyzed using Alexander's staining solution, as previously described (Alexander 1969). Pollen from flowers of Col-0 or heterozygous *erd1a* mutants (*ERD1A*/*erd1a*) in the *quartet* (*qrt*) genetic background was released on a slide with the staining solution and analyzed under a light microscope. The *qrt* mutant produces tetrad pollen in which the 4 microspores fail to separate (Rhee and Somerville 1998). Pollen from homozygous *erd1a/erd1a* plants was released by pressing the pollen sac with tweezers on a slide.

Pollen germination and tube elongation were analyzed as previously described with slight modifications (Johnson-Brousseau and McCormick 2004). Pollen from the heterozygous complementation line (*pERD1A::gERD1A-*GFP/-, *erd1a*/*erd1a*) was incubated for 5 h in a medium containing 0.01% (w/v) boric acid, 5 mM CaCl_2_, 1 mM K(NO_3_), 5 mM KCl, 1 mM MgSO_4_, and 18% (w/v) sucrose, with the pH adjusted to 7.5 with KOH. The medium with the pollen was transferred to a slide and examined under a confocal microscope to differentiate the pollen genotype based on GFP fluorescence. Pollen exhibiting GFP fluorescence was considered the *erd1a* mutant complemented with *ERD1A-GFP*, whereas nonfluorescent pollen was considered the *erd1a* mutant. Pollen with pollen tubes longer than the radius of the pollen was considered to have germinated.

A more detailed description of the material and methods can be found in the Supplemental Data section.

### Accession numbers

Sequence data from ERD1 proteins and putative orthologues described in this article can be found in the GenBank/EMBL data libraries under accession numbers: *Saccharomyces cerevsisiae*, NP_010702.4; *Aspergillus nidulans*, XP_663286.1; *Arabidopsis thaliana* ERD1A, NP_568530.1; *Arabidopsis thaliana* ERD1B, NP_001318333.1; *Oryza sativa*, EEC69602.1; *Zea mays*, ONM01771.1; *Triticum aestivum*, XP_044379600.1; *Populus trichocarpa*, XP_002310465.2; *Physcomitrium patens*, XP_024384771.1; *Medicago truncatula*, XP_003597031.1; *Ostreococcus tauri*, XP_003083286.2; *Selaginella moellendorffii*, XP_024524268.1; *Chlamydomonas reinhardtii*, XP_042928479.1; *Marchantia polymorpha*, PTQ36909.1; and *Ceratopteris richardii*, KAH7422254.1.

## Supplementary Material

kiad123_Supplementary_Data

## Supplemental data

The following materials are available in the online version of this article.

**Supplemental Figure S1**. Alignment of protein sequences of ScERD1, AtERD1A, and AtERD1B.

**Supplemental Figure S2**. Unrooted phylogenetic tree of ERD1 orthologs in plants.

**Supplemental Figure S3**. Multiple sequence alignment of ERD1 orthologs in plants.

**Supplemental Figure S4**. Expression of the pERD1::gERD1A-GUS construct in *erd1a*.

**Supplemental Figure S5**. *ERD1A* and *PHT4;6* are genes with distinct functions.

**Supplemental Figure S6**. Co-localization of ERD1A-RFP and ERD1B-GFP.

**Supplemental Figure S7**. Lack of complementation of BiP secretion phenotype of the *erd1* mutant expressing the Arabidopsis ERD1A and ERD1B proteins.

**Supplemental Figure S8**. Analysis of nucleotide-sugar levels.

**Supplemental Figure S9**. The *erd1a* mutant shows no systemic changes in Pi level in whole plants.

**Supplemental Figure S10**. No evidence of embryo lethality in siliques of *erd1a*/ERD1A lines.

**Supplemental Figure S11**. Chromatograms obtained by the high-performance anion-exchange chromatography with pulsed amperometric detection (HPAEC-PAD).

**Supplemental Table S1**. RNA-seq data for the expression of *AtERD1A* and *AtERD1B*.

**Supplemental Material and Methods.**

**Supplemental Data Set S1**. Amino acid of ERD1A putative orthologues in plants and fungi.
