# Supplementary material for: cis-Golgi phosphate transporters harboring an EXS domain are essential for plant growth and development
Source: Plant Physiol. 2023 Feb 28;192(2):1000–15. doi: 10.1093/plphys/kiad123 (PMC10231389; doi:10.1093/plphys/kiad123)
Supplement: kiad123_Supplementary_Data [file kiad123_supplementary_data.pdf]

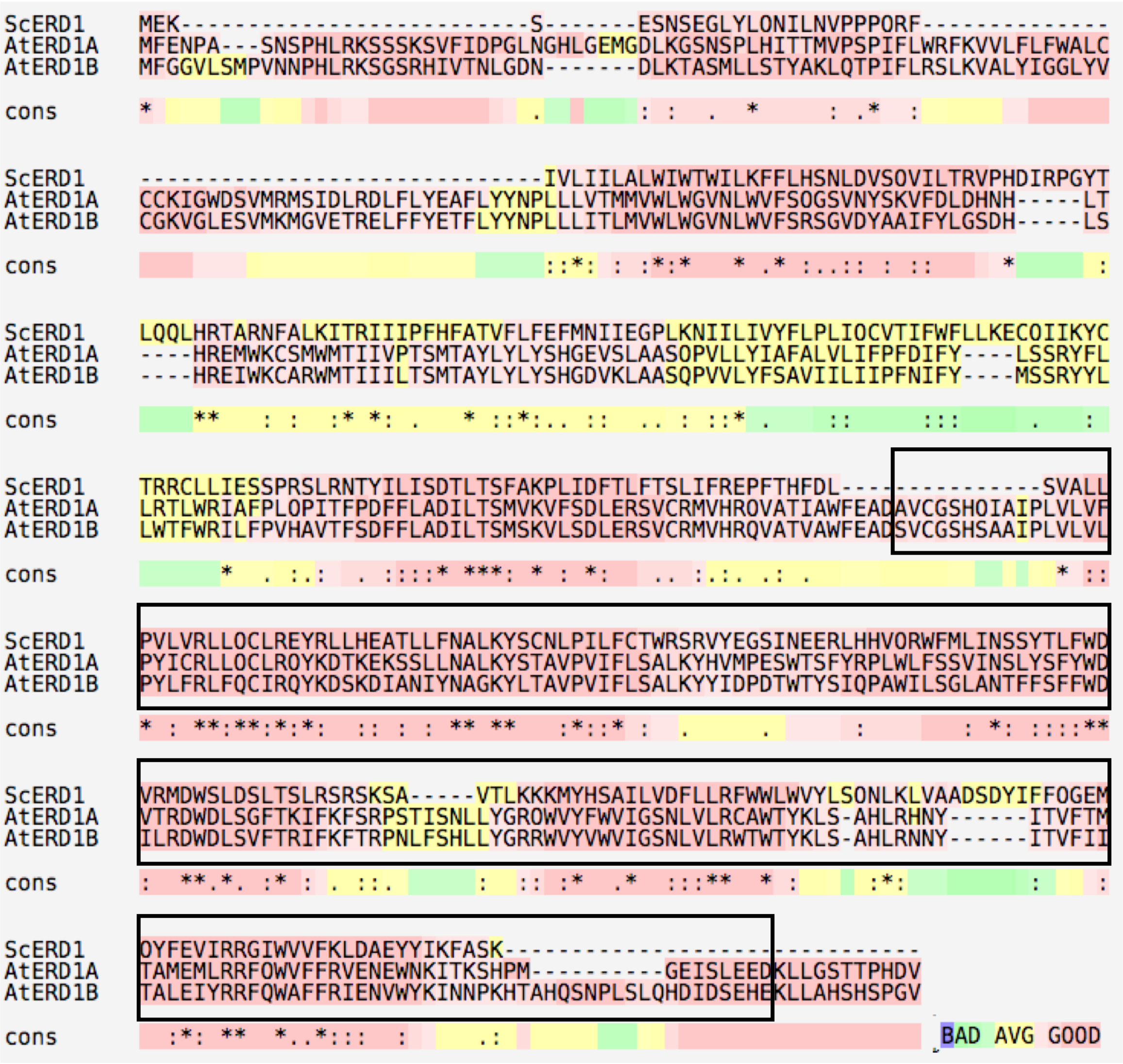

Supplemental Figure S1. Alignment of protein sequences of ScERD1, AtERD1A, and AtERD1B. Protein sequence alignment was made using T-coffee (tcoffee.crg.eu). The EXS domain is highlighted by the black box. The level of amino acid conservation is indicated with a color code as indicated in the lower right corner.

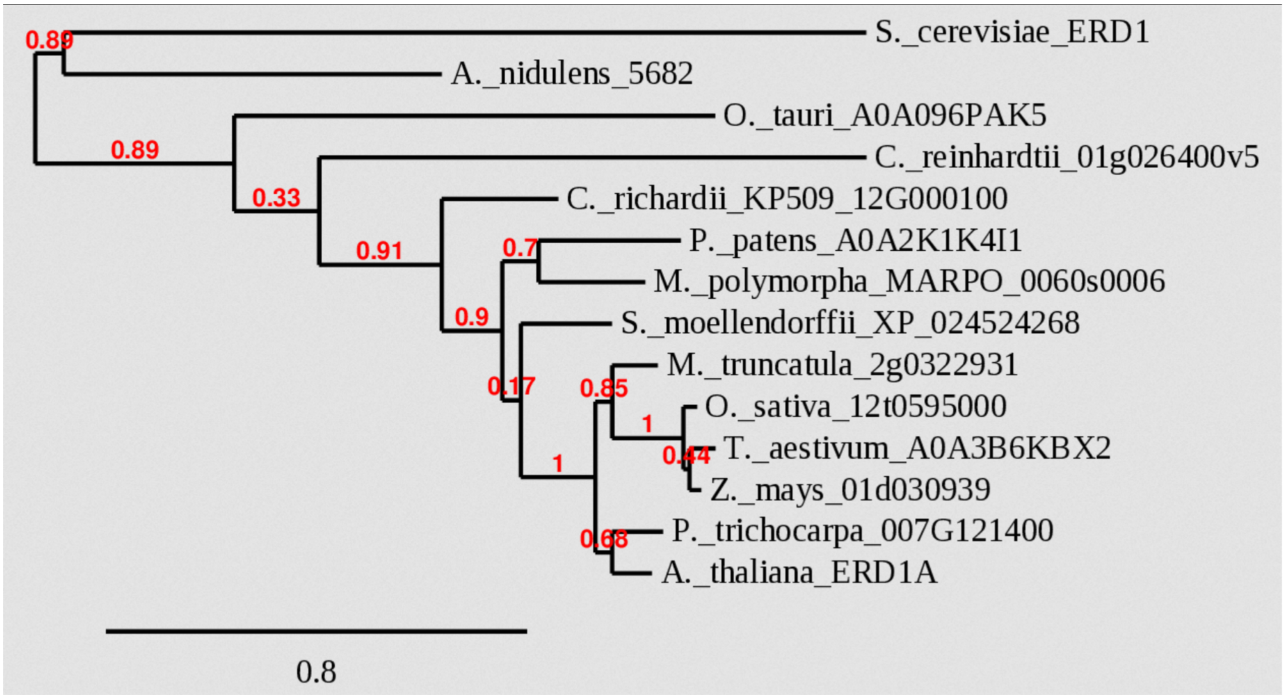

Supplemental Figure S2. Unrooted phylogenetic tree of ERD1 orthologs in plants. Numbers at the nodes indicates the bootstrap values on neighbor joining analysis. The phylogenetic tree was constructed through the MABL bioinformatics platform (https://www.phylogeny.fr). The scale bar represents the number of substitutions per site.

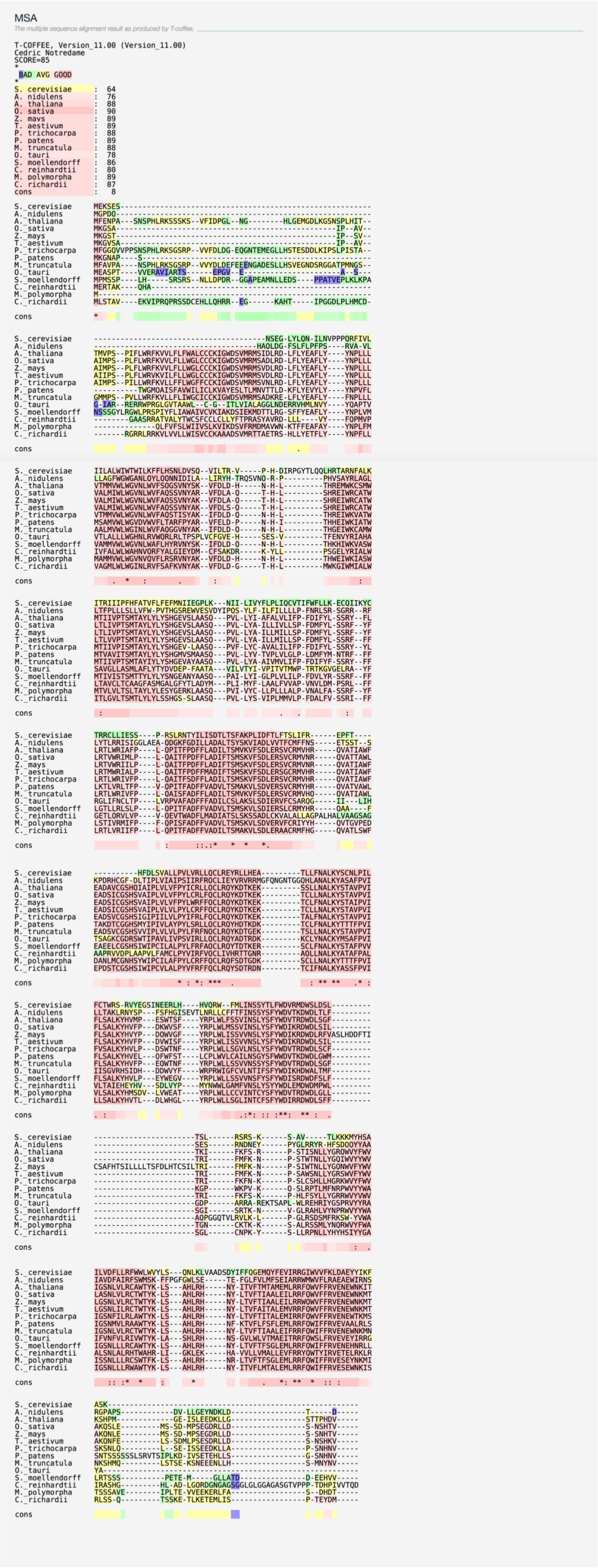

Supplemental Figure S3. Multiple sequence alignment of ERD1 orthologs in plants. Multiple sequence alignment was performed using T- coffee (tcoffee.crg.eu). The level of amino acid conservation is indicated with a color code as indicated in the upper left corner.

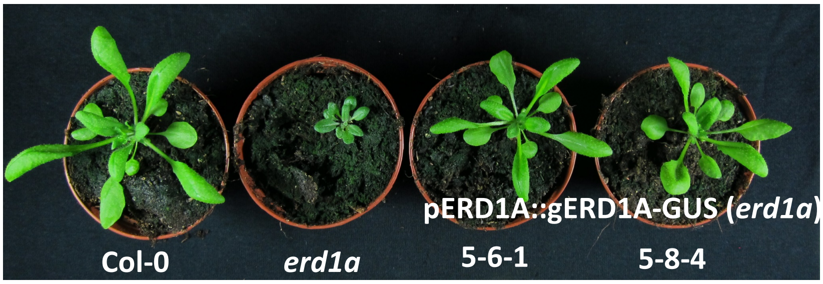

Supplemental Figure S4. Expression of the *pERD1::gERD1A-GUS* construct in *erd1a.* Expression of *pERD1::gERD1A-GUS* complements the *erd1a* mutant rosette growth phenotype.

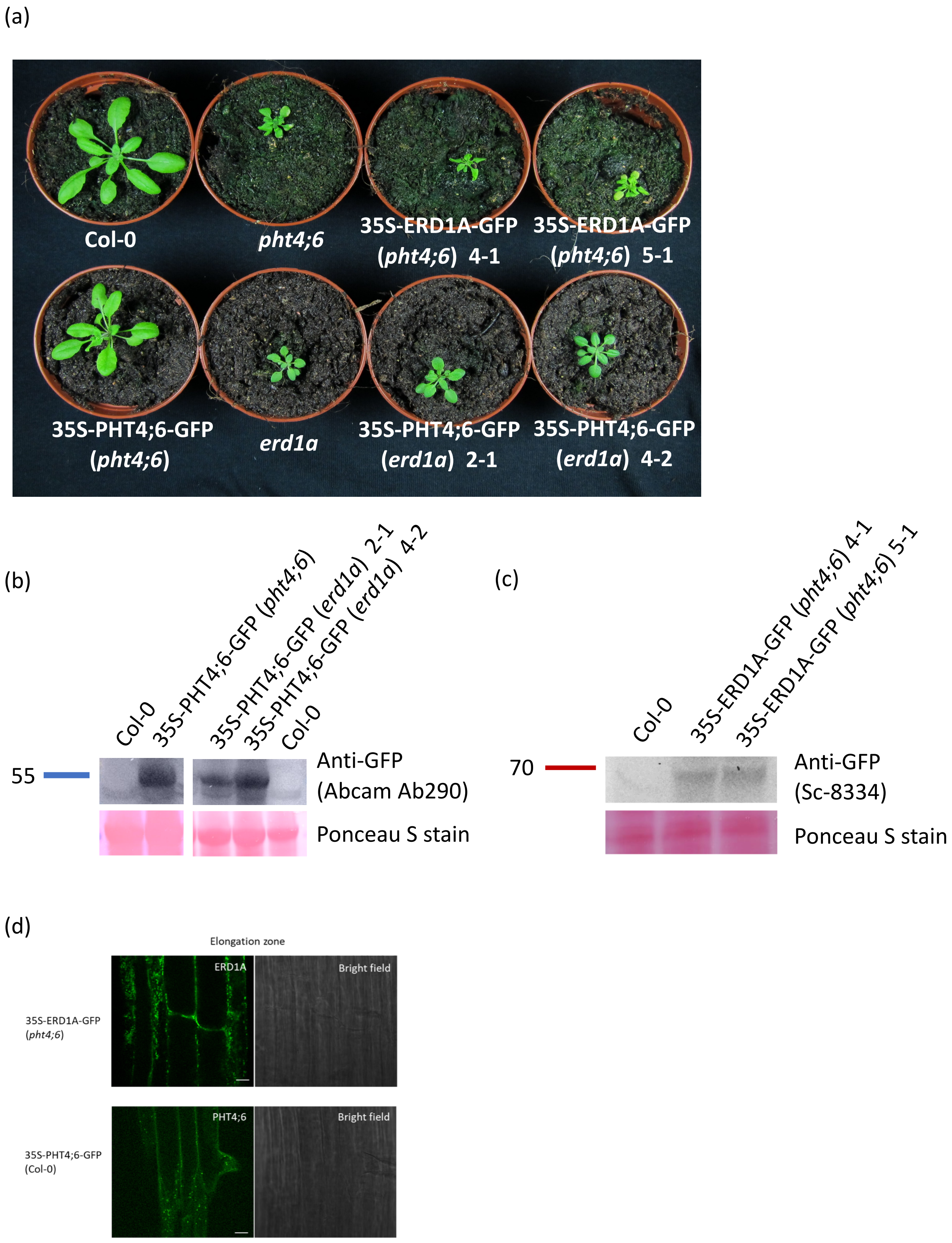

Supplemental Figure S5. ***ERD1A*** **and** ***PHT4;6*** **are genes with distinct functions.** (a) Overexpression of *ERD1A-GFP* could not complement the *pht4;6* mutant (upper row) and overexpression of *PHT4;6-GFP* could not complement the *erd1a* mutant while it complemented the *pht4;6* mutant (lower row). The expression of PHT4;6-GFP (b) and ERD1A-GFP (c) was verified by Western blot using antibodies against GFP. Confirmation of the expression of ERD1A-GFP and PHT4;6-GFP in the *pht4;6* and *erd1a* mutants, respectively, was also determined by confocal microscopy. Scale bar = 10 um.

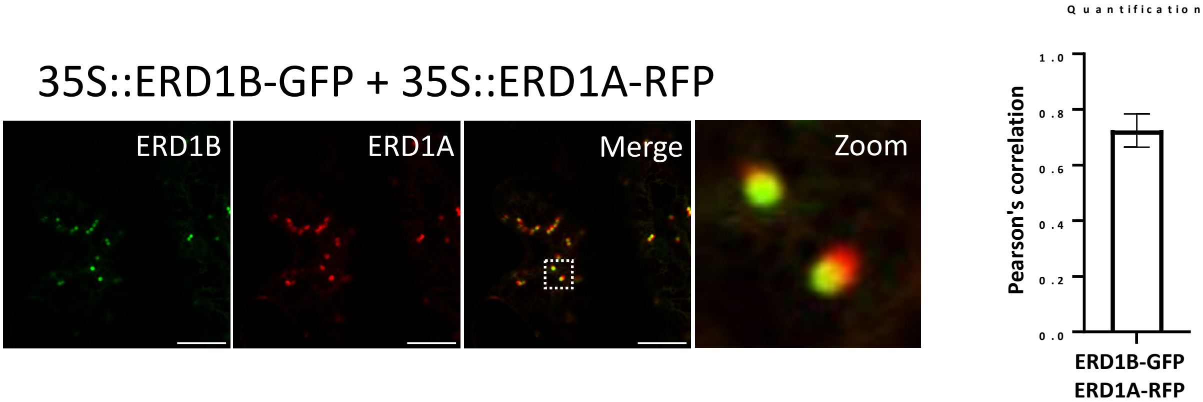

Supplemental Figure S6. Co-localization of ERD1A-RFP and ERD1B-GFP. Epidermal cells from tobacco leaf discs transiently expressing ERD1A-RFP and ERD1B-GFP show high level of co-localization. The white dashed square on the merge pictures indicated the zoom area. The Pearson's correlation indicating the colocalization level is shown on the right. Scale bars, 10 um. Error bars: ±SD.

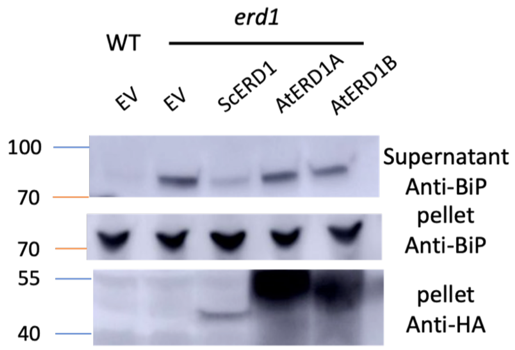

Supplemental Figure S7. Lack of complementation of BiP secretion phenotype of the *erd1* mutant expressing the Arabidopsis ERD1A and ERD1B proteins. BiP secreted in the growth media (upper lane) and present in whole cell extract (middle lane) were detected by Western blot using anti-Bip antibodies. The *S. cerevisae* ERD1 and Arabidopsis ERD1A and ERD1B expressed in the *erd1* mutant were tagged by HA at the C-terminal end and their expression was detected by Western blot using anti-HA antibodies (lower panel).

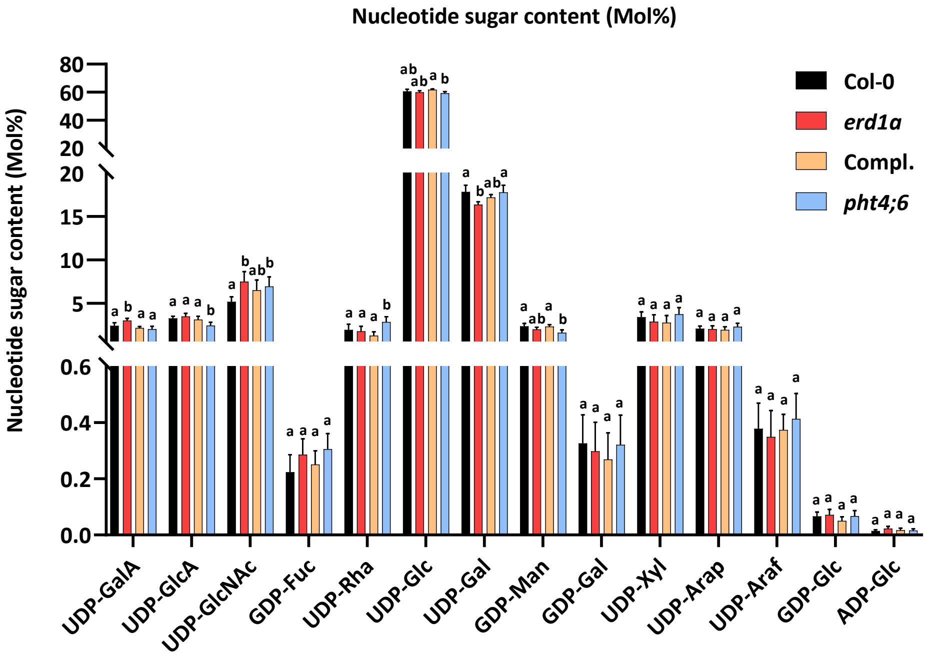

Supplemental Figure S8. Analysis of nucleotide-sugar levels. Rosettes from 4-week-old Col-0, *erd1a*, *erd1a* complemented with the *pERD1A::ERD1A-GFP* construct (Compl.) and *pht4;6* were extracted and nucleotide-sugar levels were quantified by LC-MS/MS. Statistics: one-way ANOVA and Tukey's multiple comparisons test. Error bars ±SD, $n \geq 6$, $p < 0.05$.

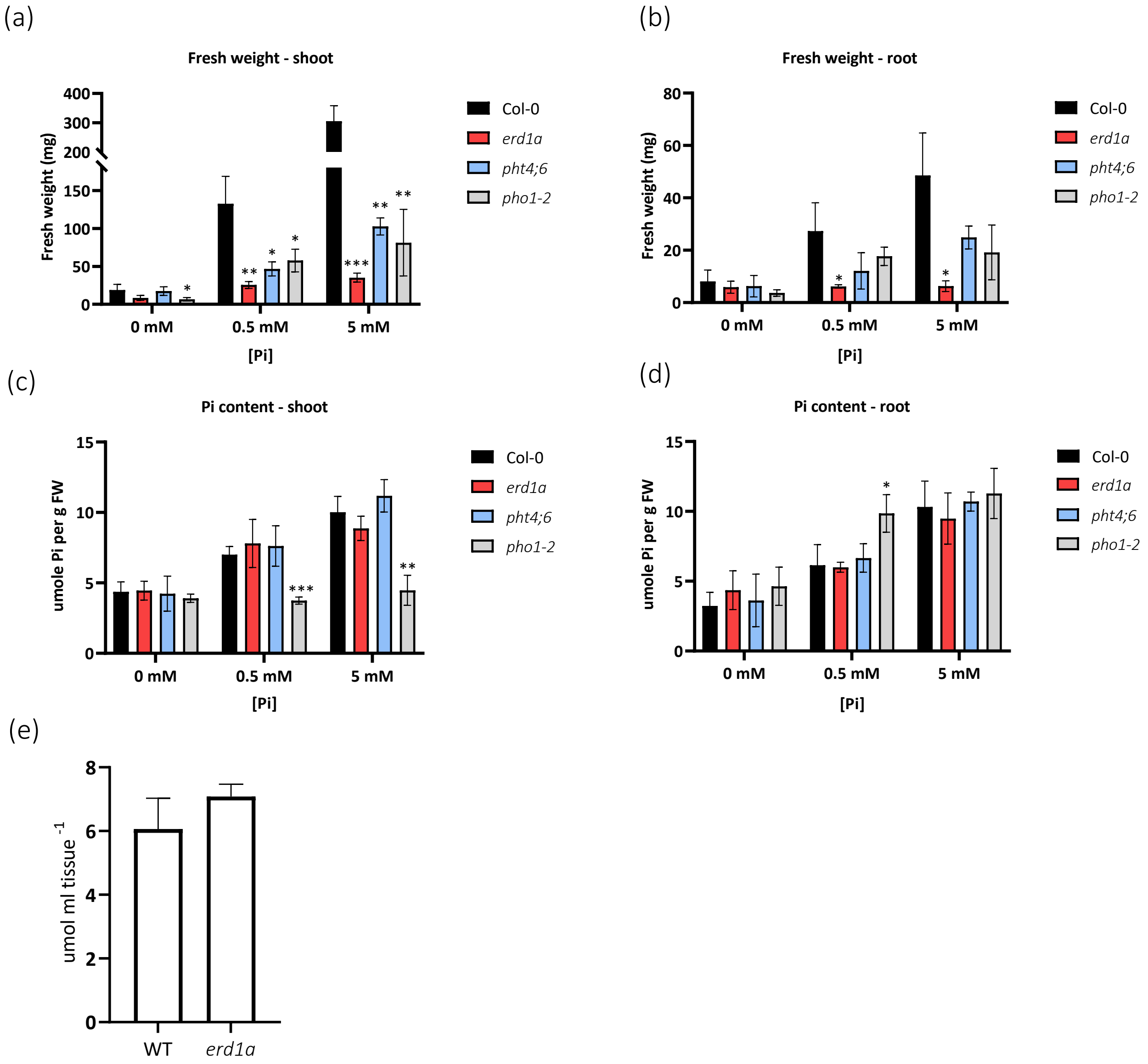

Supplemental Figure S9. The *erd1a* mutant shows no systemic changes in Pi level in whole plants. Plants were grown for 4 weeks in a soil-free clay substrate fertilized with different Pi concentrations before measuring the fresh weight of shoots (a) and roots (b) as well as the Pi content of shoots (c) and roots (d). For panels a-d, statistics represent t-test compared to Col-0 in each conditions, * $p<0.05$, ** $p<0.01$, *** $p<0.001$. Error bars: ±SD, $n \geq 3$. (e) Plants were also grown in soil for 4 weeks before measuring the vacuolor Pi content by in vivo NMR. Statistics: t-test. Error bars: ±SE, $n \geq 3$.

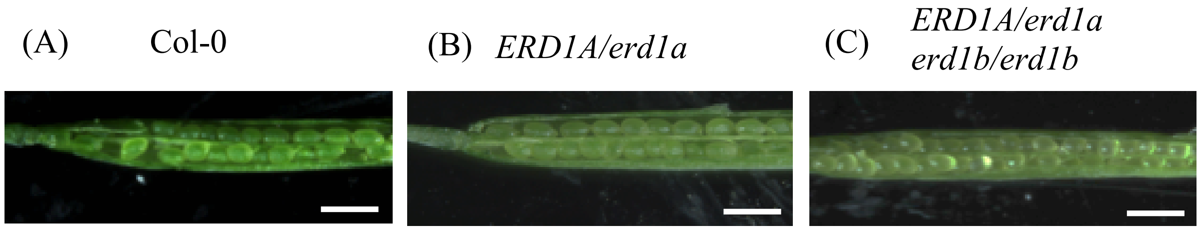

Supplemental Figure S10. No evidence of embryo lethality in siliques of *erd1a*/ERD1A lines. The genotypes of the lines analyzed are indicated above each picture. Scale bar = 1 mm.

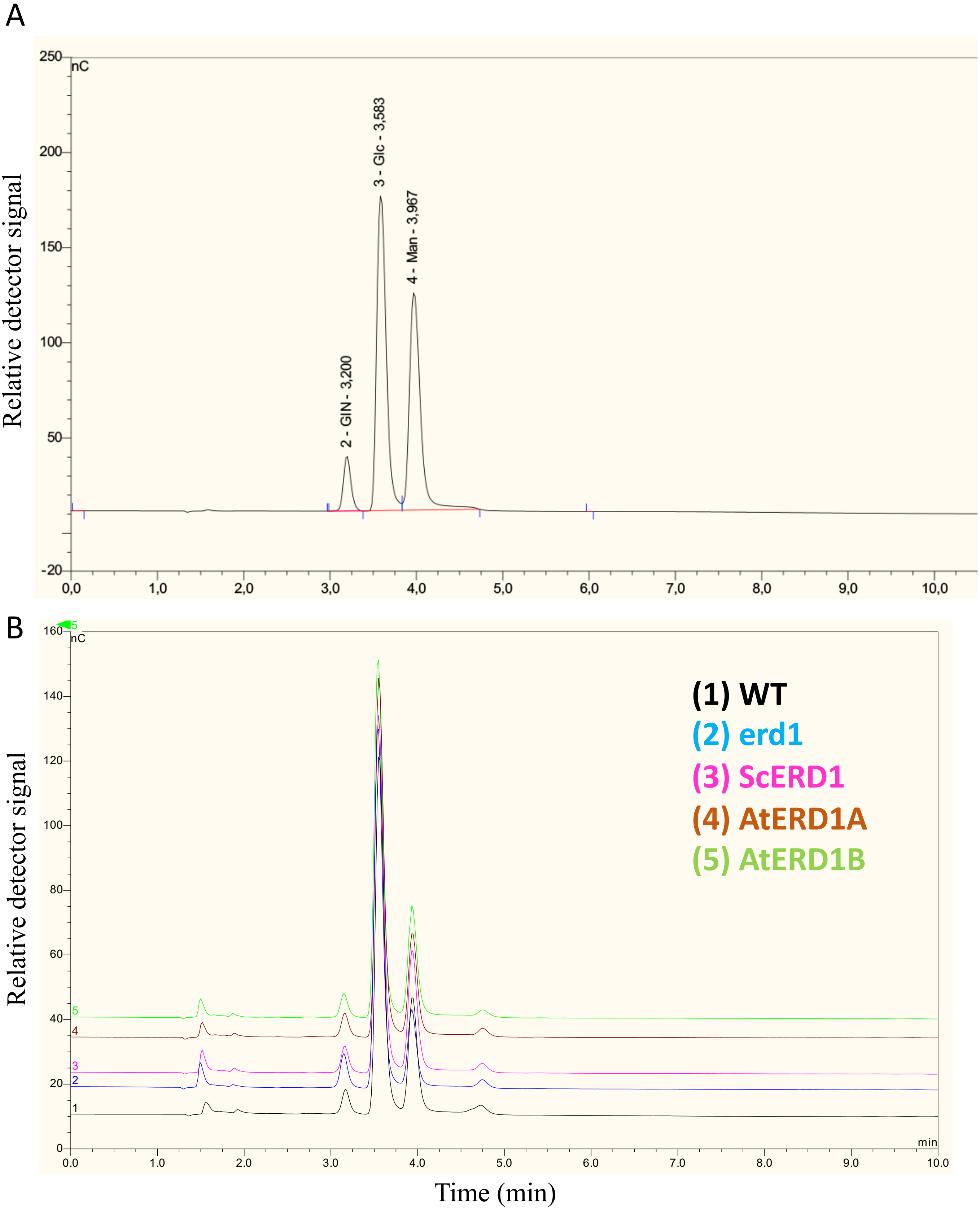

Supplemental Figure S11. Chromatograms obtained by the high-performance anion-exchange chromatography with pulsed amperometric detection (HPAEC–PAD). **A**, Standard mix of glucose (Glc, 25 µg/mL), mannose (Man, 25 µg/mL) and glucosamine (GlN, 2.5 µg/mL). **B**, Glucose, mannose and glucosamine were analysed by HPAEC-PAD after hydrolysis of yeast cell wall from WT, *erd1* mutant, and *erd1* mutant expressing *ScERD*1, *AtERD1A* and *AtERD1B*.

Supplemental Table S1. RNA-seq data for the expression of *AtERD1A* and *AtERD1B* .

Gene name	Gene code	RPKM value root	RPKM value shoot
AtERD1A	At5g35730	645	408
AtERD1B	At2g32295	169	138

The dataset was published in Desforges et al. (2019) Plant Physiol. 180, 305.
It is available at Gene Expression Omnibus database, accession no.GSE116553.

Supplemental Material and Methods

***A. thaliana* cell wall monosaccharide composition**

The cell wall material of Arabidopsis was prepared by following the previous publication with modification (Zablackis et al., 1995). The plants growing in the long-day condition were transferred to the dark for 24 hours before the mature leaves were collected. The samples were ground with liquid nitrogen and dried by freeze-drying. The alcohol-soluble fraction was removed by heating the dry materials twice with 80% EtOH and 70% EtOH at 95°C for 30 minutes. The alcohol-insoluble residues 1 (AIR1) pellets were collected by centrifugation at 14000 rpm for 10 minutes and washed with chloroform:MeOH (1:1) and 100% acetone solutions before dried by Speedvac vacuum concentrator. The dried AIR1 was resuspended in buffer containing 0.1 M potassium phosphate buffer pH 7.0 and 0.01% sodium azide (NaN3) and treated with alpha-amylase (Roche) for digestion of starch at 37°C for 24 hours twice. The pellet was washed several times with buffer, water, and acetone before dried by Speedvac vacuum concentrator. The cell wall monosaccharide analysis using the trimethylsilyl (TMS) derivatisation method was done with the resulting cell wall materials (AIR2) in the Biopolymer Analytical Platform (Umea Plant Science Center,Sweden). 500 ug (± 10%) dry fine powder of AIR2 and 30 ug of inositol, used as internal standard, together with standards of nine monosaccharides (Ara, Rha, Fuc, Xyl, Man, Gal, Glc, GalA and GlcA, each at 5, 10, 20, 50 and 100 ug) were methanolysed and derivatised, and its silylated monosaccharides were separated in GC/MS (7890A/5975C; Agilent Technologies , Santa Clara, CA, USA) (Gandla et al., 2015).

Raw data MS files from GC/MS analysis were converted to CDF format in Agilent Chemstation Data Analysis (Version E.02.00.493) and exported to R software (Version 3.0.2) (R Development Core Team, R Foundation for Statistical Computing, Vienna, Austria). Data pretreatment procedures, such as baseline correction and chromatogram alignment, time-window setting and multivariate curve resolution (MCR) processing, followed by peak identification was performed in R. 4-*O*-methylglucuronic acid was identified as previously published (Chong et al., 2013).

A. thaliana crystalline cellulosic content

For crystalline cellulose measurements, alcohol-insoluble residue (AIR) was extracted from rosette leaves, ground using a ball mill and sequentially washed with 70% ethanol, chloroform:methanol (1:1 v/v), and acetone. After enzymatically removing starch overnight (Polko et al., 2018), 350 µg aliquots of each de-starched AIR samples were subjected to the two-step sulfuric acid hydrolysis and analyzed as previously described (Mielke et al., 2021). In brief, carbohydrates were profiled on a high-performance anion-exchange chromatography with pulsed amperometric detection (HPAEC-PAD) on a 940 Professional IC Vario ONE/ChS/PP/LPG instrument (Metrohm) equipped with Metrosep Carb2 250/4.0 analytical and guard columns. Crystalline glucose was quantified as the difference between replicates with or without a 72% (w/w) sulfuric acid pretreatment at room temperature, before Seaman hydrolysis [4% (w/w) sulfuric acid at 120°C for 60 min].

^{31}P-NMR (nuclear magnetic resonance)

The *in vivo* ^{31}P-NMR analyses were performed using a standard broad-band 10 mm probe on a Bruker AMX 600 spectrometer (Bruker Analytische Messtechnik, Ettlingen, Germany) equipped with TopSpin software version 1.3. The ^{31}P-NMR spectra were recorded at 242.9 MHz without lock, using Waltz-based broad-band proton decoupling and a spectral window of 16 kHz. The spectra were acquired using a 90° pulse angle with a 6-s recycle time and were the sum of 1000 scans. Leaves previously infiltrated in the perfusion medium under a gently vacuum were packed into a 10 mm diameter NMR tube equipped with a perfusion system through which the aerated thermo-regulated (25°C) medium (1 mM methylene diphosphonate, 1mM Mes/Bis-Tris propane, pH 6.1, 0.4 mM $CaSO_4$) flowed at 10 ml min^{-1}.

Resonances were assigned as previously described (Roberts et al., 1980; Kime et al., 1982).. Phosphate concentration in the vacuole was by referring the resonance intensity with that of a glass capillary containing 33 mM methylene diphosphonate, that was previously calibrated against standard solutions (Spickett et al., 1992).

Western blotting

Seedlings growing on 1/2MS plates 7-days after germination (DAG) were collected and frozen in liquid nitrogen. Tissues were lysed with the TissueLyser and incubated in lysis buffer

containing 120 mM Tris-HCl pH8.8, 1% SDS, 10% glycerol, and protease inhibitor cocktail (Sigma) on ice for 30 min. Samples were heated at 70°C for 10 min with loading dye(12 mM Tris-HCl pH6.8, 1.2% SDS, 1% glycerol, 2.2% 2-mercaptoethanol, and 0.002% bromophenol blue). Commercial SDS-PAGE (GenScript) was used to run the gel and 0.45 um PVDF membranes (Bio-Rad) were used for transfer. Primary antibodies anti-GFP (Santa Cruz Biotechnology, Sc-8334 or Abcam ab290) and anti-HA (sigma, 3F10 monoclonal) were utilized.

RNA preparation and RT-qPCR

Mature leaves or flowers from 4-5-week-old plants or 7-DAG seedlings growing on 1/2MS plates were harvested and frozen in liquid nitrogen before grinding with glass beads bythe TissueLyser (Qiagen). The isolated RNA (Oñate-Sánchez *et al.*, 2008) was used for cDNA synthesis by M-MLV reverse transcriptase (Promega). Q-RT-PCR was performed with SYBR-select master mix (Thermo) and QuantStudio (Thermo) by following the manufacturer's manual.

Protoplasting of *N. benthamiana* and GFP localisation

The leaves of 4-5 week old *N. benthamiana* were infiltrated with *A. tumefaciens* transformed with the appropriate plasmid. To generate the mesophyll protoplast, the infiltrated leaves were cut into small strips and incubated in the enzyme solution containing 1% (w/v) cellulase Onozuka R10, 0.5 % (w/v) macerozyme Onozuka R10, 20 mM MES (pH 5.8), 0.5 M mannitol, 20 mM KCl, and 10 mM $CaCl_2$ with shaking at 40 rpm for two hours at room temperature. The solution with suspended protoplasts was spun down at 100 xg at 4°C and resuspended in the buffer containing 154 mM NaCl, 125 mM $CaCl_2$, 5 mM KCl, and 0.5 M MES (pH 5.6). All the images were observed by confocal microscopy Leica Stellaris. The quantification of colocalization correlation was analyzed by ImageJ with the JACoP plugin.

References

Chong SL, Koutaniemi S, Virkki L, Pynnonen H, Tuomainen P, Tenkanen M (2013) Quantitation of 4-O-methylglucuronic acid from plant cell walls. Carbohydrate Polymers **91:** 626-630

Gandla ML, Derba-Maceluch M, Liu XK, Gerber L, Master ER, Mellerowicz EJ, Jonsson LJ (2015) Expression of a fungal glucuronoyl esterase in Populus: Effects on wood properties and saccharification efficiency. Phytochemistry **112:** 210-220

Kime MJ, Ratcliffe RG, Loughman BC (1982) THE APPLICATION OF P-31 NUCLEAR MAGNETIC-RESONANCE TO HIGHER-PLANT TISSUE .2. DETECTION OF INTRACELLULAR CHANGES. J Exp Bot **33:** 670-681

Mielke S, Zimmer M, Meena MK, Dreos R, Stellmach H, Hause B, Voiniciuc C, Gasperini D (2021) Jasmonate biosynthesis arising from altered cell walls is prompted by turgor-driven mechanical compression. Science Advances **7**

Polko JK, Barnes WJ, Voiniciuc C, Doctor S, Steinwand B, Hill JL, Tien M, Pauly M, Anderson CT, Kieber JJ (2018) SHOU4 proteins regulate trafficking of cellulose synthase complexes to the plasma membrane. Curr Biol **28:** 3174-+

Roberts JKM, Ray PM, Wadejardetzky N, Jardetzky O (1980) ESTIMATION OF CYTOPLASMIC AND VACUOLAR PH IN HIGHER-PLANT CELLS BY P-31 NMR. Nature **283:** 870-872

Spickett CM, Smirnoff N, Ratcliffe RG (1992) Metabolic response of maize roots to hyperosmotic shock - an in vivo p-31 nuclear-magnetic-resonance study. Plant Physiol **99:** 856-863

Zablackis E, Huang J, Muller B, Darvill AG, Albersheim P (1995) Characterization of the cell-wall polysaccharides of *Arabidopsis thaliana* leaves. Plant Physiol **107:** 1129-1138

Supplemental Dataset 1
Amino acid sequence of ERD1A putative orthologues in plants and fungi

>S._cerevisiae_ERD1
MEKSESNSEGLYLQNILNVPPPQRFIVLIILALWIWTWILKFFLHSNLDVSQVILTRVPHDIRPGYTLQQLHR
TARNFALKITRIIIPFHFATVFLFEFMNIIEGPLKNIILIVYFLPLIQCVTIFWFLLKECQIIKYCTRRCLLIESSPRSL
RNTYILISDTLTSFAKPLIDFTLFTSLIFREPFTHFDLSVALLPVLVRLLQCLREYRLLHEATLLFNALKYSCNLPIL
FCTWRSRVYEGSINEERLHHVQRWFMLINSSYTLFWDVRMDWSLDSLTSLRSRSKSAVTLKKKMYHSAIL
VDFLLRFWWLWVYLSQNLKLVAADSDYIFFQGEMQYFEVIRRGIWVVFKLDAEYYIKFASK

>A._nidulens_5682
MGPDQHAQLDGFSLFLPFPSRVAVLLLAGFWGWGANLQYLQQNNIDILALIRYHTRQSVNQRPPHVSA
YRLAGLLTFPLLLSLLVFWPVTHGSREWVESVDYIPQSYLFILFILLLLPFNRLSRSGRRRFLYTLRRISIGGLAE
AQDGKFGDILLADALTSYSKVIADLVVTFCMFFNSETSSTSKPDRHCGFDLTIPLVIAIPSIIRFRQCLIEYVRV
RRMGFQNGNTGGQHLANALKYASAFPVILLTAKLRNYSPFSFHGISEVTLNRLLCFFTFINSSYSFYWDVTK
DWDLTLFSESRNDNEYPYGLRRYRHFSDQQYYAAIAVDFAIRFSWMSKFFPGFGWLSETEFGLFVLMFSE
IARRWMWVFLRAEAEWIRNSRGPAPSDVLLGEYNDKLDTD

>A._thaliana_ERD1A
MFENPASNSPHLRKSSSKSVFIDPGLNGHLGEMGDLKGSNSPLHITTMVPSPIFLWRFKVVLFLFWALCC
CKIGWDSVMRMSIDLRDLFLYEAFLYYNPLLLVTMMVWLWGVNLWVFSQGSVNYSKVFDLDHNHLTH
REMWKCSMWMTIIVPTSMTAYLYLYSHGEVSLAASQPVLLYIAFALVLIFPFDIFYLSSRYFLLRTLWRIAFP
LQPITFPDFFLADILTSMVKVFSDLERSVCRMVHRQVATIAWFEADAVCGSHQIAIPLVLVFPYICRLLQCL
RQYKDTKEKSSLLNALKYSTAVPVIFLSALKYHVMPESWTSFYRPLWLFSSVINSLYSFYWDVTRDWDLSG
FTKIFKFSRPSTISNLLYGRQWVYFWVIGSNLVLRCAWTYKLSAHLRHNYITVFTMTAMEMLRRFQWVFF
RVENEWNKITKSHPMGEISLEEDKLLGSTTPHDV

>O._sativa_12t0595000
MKGSAIPAVAIMPSPLFLWRFKVVLFLLWGLCCCKIGWDSVMRMSADLRDLFLYEAFLYYNPLLLVALMI
WLWGVNLWVFAQSSVNYARVFDLAQTHLSHREIWRCATWLTLIVPTSMTAYLYLYSHGEVSLAASQPV
LLYAILLIVLLSPFDMFYLSSRFYFLRTVWRIMLPLQAITFPDFFLADIFTSMSKVFSDLERSVCRMVNRQVA
TTAWLEADSICGSHSVAIPLVLVFPYLCRLFQCLRQYKDTKEKTCLLNALKYSTAVPVIFLSALKYHVFPDKW
VSFYRPLWLMSSVINSLYSFYWDIKRDWDLSILTRIFMFKNPSTWTNLLYGQIWVYYWVLASNLVLRCTW
TYKLSAHLRHNYLTVFTIAALEILRRFQWVFFRVENEWNKMTAKQSLEMSSDMPSEGDRLLDSNSHTV

>Z._mays_01d030939
MKGSTIPSVAIMPSPLFLWRFKVVLFLLWGLCCCKIGWDSVMRMSVDLRDLFLYEAFLYYNPLLLVALMI
WLWGVNLWVFAQSSVNYAKVFDLAQTHLSHREIWRCATWLTLIVPTSMTAYLYLYSHGEVSLAASQPVL
LYAILLMILLSPFDMFYLSSRFYFLRTVWRIILPLQAITFPDFFLADIFTSMSKVFSDLERSVCRMVNRQVATI
AWFEADSICGSHSVAIPLVLVFPYLWRFFQCLRQYKDTKEKTCLFNALKYSTAIPVIFLSALKYHVYPDQWV
GFYRPLWLISSVVNSLYSFYWDIKRDWDLRFVASLHDDFTICSAFHTSILLLLTSFDLHTCSILTRIFMFKNPSI
WTNLLYGQNWVFYWVLGSNLVLRCTWTYKLSAHLRHNYLTVFTIAALEILRRWQWVFFRVENEWNKM
TAKQNLEMSSDMPSEGDRLLDSSNHTV

>T._aestivum_A0A3B6KBX2
MKGVSAIPAVAIIPSPLFLWRVKVILFLLWGLCCCKIGWDSVMRMSVDLRDLFLYEAFLYYNPLLLVALMI
WLWGVNLWVFAQSSVNYAKVFDLSQTHLSHREIWRCATWLTLVVPTSMTAYLYLYSHGEVSLAASQPV

LLYAILLMILLSPFDMFYLSSRFFFLRTMWRIALPLQAITFPDFFMADIFTSMSKVFSDLERSGCRMVHRQV
ATIAWFEADSICGSHSVAIPLVLVLPYLCRFFQCLRQYKDTKEKTCLLNALKYSTAVPVIFLSALKYHVFPDV
WISFYRPLWLISSVVNSLYSFYWDIKRDWDLSILTRIFMFKNPSAWSNLLYGRSWVFYWVLGSNLILRCT
WTYKLSAHLRHNYLTVFAITALEMVRRFQWVFFRVENEWNKMTAKQNFELSSDMLPSESDRLLDSNSH
KV

>P._trichocarpa_007G121400
MFGGQVVPPSNSPHLRKSGSRPVVFDLDGEQGNTEMEGLLHSTESDDLKIPSLPISTAAIMPSPILLWRFK
VLLFFLWGFFCCKIGWDSVMRMSVNLRDLFLYEAFLYYNPLLLVTMMVWLWGVNLWVFAQSTISYAKIF
DLDQNHLTHREIWKVATWMTIIVPISMTAYLYLYSHGEVLAASQPVFLYCAVALILIFPFDIFYLSSRYYLLRT
LWRIVFPLQAITFSDFFLADILTSMSKVFSDLERSVCRMVHRQVATIAWFEADSVCGSHSIGIPIILVLPYIFR
LFQCLRQYKDTKEKTALFNALKYSTAVPVIFVSALKYHVLPDSWTNFYRPLWLLSGVLNSLYSFYWDVTRD
WDLSCFTRIFKFNKPSLCSHLLHGRKWVYFWVIGSNFILRLAWTYKLSAHLRHNYLTVFTITALEMIRRFQ
WVFFRVENEWTKMSSKSNLQLSEISSEEDKLLAPSNHNV

>P._patens_A0A2K1K4I1
MKGNAPSTWGMQAISFAVWILICLKVAYESLTLMNVTTLDKFLYEVYLYYNPVFLMSAMVWLWGVDV
WVFLTARFPYARVFELDPNHITHHEIWKIATWMTVAVITSMTAYLYLYSHGMVSMAASQPVLLYVTVPL
VLGLPLDMFYMNTRFFFLKTLVRLTFPVQPITFADFFVADVLTSMAKVLSDVERSVCRMYHRQVATVAW
LTAKDTCGGHSMYIPIVLAYPYLSRLLQCLRQYHDTKDKTCLFNALKYTTTFPVIFLSALKYHVELQFWFSTL
CPLWVLCAILNSGYSFWWDVTKDWDLGWMKGPWKPVKQSLRPTLMFNRPWVYYWAIGSNMVLRA
AWTYKLSAHLRHNFKTVFLFSFLEMLRRFQWIFFRVEVAALRLSSNTSSSSSSLSRVTSIPLKDIVSETEHLLS
GSNHNV

>M._truncatula_2g0322931
MFAVPANSPHLRKSGSRPVVYDLDEFEEENGADESLLHSVEGNDSRGGATPMNGSGMMPSPVLLWRF
KVLLFLIWGCICCKIGWDSVMRMSADKRELFLYEAFLYFNPLLLAALMVWLWGINLWVFAQGGVNYAKI
FDLDQNHLTHGEIWKCAMWMTIIVPTSMTAYIYLYSHGEVAYAASQPVLLYAAIVMVLIFPFDIFYFSSRY
FFLRTLWRIVFPLQAISFADFFLADILTSMVKVFSDLERSVCRMVHQQVATIAWLEADSVCGSHSVVIPLVL
VLPYLFRFNQCLRQYKDTGEKTSLLNALKYSTAVPVIFLSALKYHVFPEQWTNFYRPLWLLSSVVNSSYSFY
WDVTRDWDLSGFTRIFKFSKPHLFSYLLYGRRWVYVWVIGSNLVLRCTWTYKLSAHLRHNYLTVFTIAALE
IFRRFQWIFFRVENEWNKMNNKSHMQLSTSEKSNEEENLLHSMNYNV

>O._tauri_ A0A096PAK5
MEASPTVVERAVIARTSEPGVEASGIARRERRWPRGLGVTAAWLCGITLVIALAGGLNDERRVHMLNVY
YQAPTVVTLALLLWGHNLRVWQRLRLTPSPLVCFGVEHSESVTFENVYRIAHASAVGLLASMLAFLYTYD
VDEPFAATAVILVTYIVPITVTMWPTRTKGVGELRAYFRGLIFNCLTPLVRPVAFADFFFADILCSLAKSLSDI
ERVFCSARQGIILIHTSAGKCGDRSWTIPAVLIVPSVIRLLQCLRQYADTRDKKCLYNACKYMSAFPVIIISGV
RHSIDHDDWVYFWRPRWIGFCVLNTIFSFYWDIKHDWALTMFGDPARRAREKTSAPLWLREHRIYGSP
RVYYRAIFVNFVLRIVWTYKLASHLRHNSGVLWLVTMAEITRRFQWSLFRVEVEYIRRGYA

>S._moellendorffii_XP_024524268
MPMSSPLHSRSRSNLLDPDRGGAPEAMNLLEDSPPATVEPLKLKPANSSSGYLRGWLPRSPIYFLIAWAIV
CVKIAKDSIEKMDTTLRGSFFYEAFLYYNPLVMVAMMVWLWGVNLWAFLHYRVNYSKIFDLDQNHLTH
KHIWKVASWMTIVISTSMTTYLYLYSNGEANYAASQPAILYIGLPLVLILPFDVLYRSSRFFFLGTLLRLSLPL
QPITFADFFVADVLTSMSKVLSDIERSLCRMYHRQAAFEAEELCGSHSIWIPCILALPYLFRFAQCLRQYTD
TKERSCLFNALKYSTAFPVVFLSALKYHVLPEYWEGVYRPLWLLSSVVNSFYSFYWDISRDWDFSLFSGISR

TKNVGLRAHLVYNPRWVYYWAIGSNLLLRCAWTYKLSAHLRHNYLTVFTFSGLEMLRRFQWIFFRVENE
HNRLLLRTSSSPETEMGLLATDDEEHVV

>C._reinhardtii_01g026400v5
MERTAKQHAGAASRRATVALYTWCSFCCLCLLYFTPRASYAVRDLLLVYFQPMVPIVFALWLWAHNVQ
RFYALGIEYDMCFSAKDRKYLLPSGELYRIALWLTAVCLTCAAGFASMGALGFYTLADYMPLIMYFLAALF
VVAPVNVLDMPSRLFFGETLQRVLVPVQEVTWADFLMADIATSLSKSSADLCKVALALLAGPALHALVAA
GSAGAAPRVVDPLAAPVLFAMCLPYVIRFVQCLIVHRTTGNRAQLLNALKYATAFPALVLTAIEHEYHVSD
LVYPMYNWWLGAMFVNSLYSYYWDLEMDWDMPWLAQPGGQTVLRVLKLPGLRSDSMFRKSWYVW
AALSNLALRHTWAHRLIGKLEKHAVVLLVMALLEVFRRYQWTYIRVETELRKLRIRASHGHLADLGQRDG
NGAGSGGLGLGGAGASGTVPPPTDHPIVVTQD

>M._polymorpha_ MARPO_0060s0006
MQLFVFSLWIIVSLKVIKDSVFRMDMAVWNKTFFEAFAYYNPLFMMAMMVWLWGVNVQVFLRSRVN
YAKVFDLDHNHLTHWEIWKIASWMTVLVLTSLTAYLYLESYGERKLAASQPVIVYCLLPLLLALPVNALFAS
SRFYFLSTIVRMIFPFQPISFADFFVADVLTSMSKVLSDVERVFCRIYYHQVTGVPEDDANLMCGNHSYWI
PCILAFPYLCRFFQCLRQFSDTGDKSCLLNALKYTTTFPVIVLSALKYHMSDVLVWEATYRPLWLLCCVINT
CYSFYWDVTRDWDLGLLTGNCKTKKSALRSSMLYNQRWVYFWAISSNLLLRCSWTFKLSAHLRHNRLTV
FTFSGLEMLRRFQWIFFRVESEYNKMITSSSAVEIPLTEVVEEKERLFASDHDT

>C._richardii_KP509_12G000100
MLSTAVEKVIPRQPRSSDCEHLLQHRREGKAHTIPGGDLPLHMCDRGRRLRRKVLVVLLWISVCCKAAAD
SVMRTTAETRSHLLYETFLYYNPFLLVAGMLWLWGINLRVFSAFKVNYAKVFDLDGTHLMWKGIWMIAL
WITLGVLTSMTLYLYLSSHGSSLAASQPVLLYSVIPLMMVLPFDALFVSSRIFFLRTLVRIIFPLQPITFADFFV
ADILTSMAKVLSDLERAACRMFHGQVATLSWFEPDSTCGSHSIWIPCVLALPYVFRFFQCLRQYSDTRDN
TCIFNALKYASSFPVILLSALKYHVTLDLWHGLYRPLWLLSGLINTCFSFYWDIRRDWDLSFFSGLCNPKYSL
LRPNLLYHYHSIYYGAIGSNLLLRWAWTYKLSAHLRHNYITVFLMTALEMLRRFQWIFFRVESEWNKISRL
SSQTSSKETLKETEMLISPTEYDM
